# T6SS1 suppresses pro-inflammatory cytokine transcription to drive immune evasion and systemic infection in *Vibrio parahaemolyticus*

**DOI:** 10.1128/iai.00587-25

**Published:** 2025-12-05

**Authors:** Shuqi Lu, Shuo Yuan, Pengxuan Liu, Xuerui Bai, Quan Zhang, Lanfang Kong, Xiangan Han, Wei Jiang

**Affiliations:** 1 Shanghai Veterinary Research Institute, Chinese Academy of Agricultural Sciences 118161 , Shanghai, China; Stanford University School of Medicine, Stanford, California, USA

**Keywords:** *Vibrio parahaemolyticus*, type VI secretion system 1 (T6SS1), virulence, NF-κB pathway, immune evasion

## Abstract

The type VI secretion system (T6SS) is a major virulence factor in *Vibrio parahaemolyticus*, but its pathogenic mechanisms are poorly understood or still not fully understood. This study investigates how two critical T6SS1 structural components, VipA1 and Hcp1, contribute to bacterial virulence and host inflammatory responses. Comparative proteomics revealed 149 secreted proteins dependent on T6SS1, including 28 core proteins requiring both VipA1 and Hcp1 for secretion. These proteins were functionally linked to metabolic pathways such as folate-mediated one-carbon metabolism and lysine degradation, as well as structural processes like flagellar assembly. Phenotypic analysis revealed that the Δ*vipA1-hcp1* double mutant showed markedly attenuated virulence: 52.7% reduction in antibacterial activity compared to the wild-type strain. Biofilm formation increased 2.1-fold at 30°C and 2.8-fold at 37°C in Δ*vipA1-hcp1*, while swimming and swarming motility decreased by 30.9% and 35.5%. *In vivo*, Δ*vipA1-hcp1* caused only 50% mortality in mice, compared to 91.7% for the wild-type strain, and exhibited 3- to 15-fold lower bacterial loads in the blood, liver, and spleen. Histopathological analysis confirmed that the Δ*vipA1-hcp1* failed to induce tissue damage, unlike the wild-type strain. At the host interface, deletion of *vipA1* and *hcp1* led to significantly elevated inflammatory cytokine (IL-1β, IL-8, and IL-6) mRNA levels. Mechanistically, T6SS1 inhibited NF-κB activation by stabilizing IκBα and reducing p65 nuclear translocation (40.0% in wild-type-infected cells vs 85.8% in double mutant-infected cells). These findings establish VipA1 and Hcp1 as critical regulators of T6SS1-mediated coordinating effector secretion, virulence, immune evasion, and lethality, providing novel mechanistic insights into *V. parahaemolyticus* pathogenesis.

## INTRODUCTION

*Vibrio parahaemolyticus* is a gram-negative marine bacterium and a leading global cause of seafood-borne acute gastroenteritis, as well as more severe infections like wound infections and septicemia (1, 2). Its virulence is mediated through an arsenal of pathogenic determinants, notably the type III secretion system (T3SS), the type VI secretion system (T6SS), and adhesins, among others (3).

Structurally analogous to bacteriophage tail complexes, the T6SS is phylogenetically widespread, present in about a quarter of gram-negative bacterial genomes (4). T6SS depends on 13 fundamental and conserved core components for the secretion of effector proteins into target cells (5). The VipA (TssB) and VipB (TssC) proteins assemble into tubular structures that closely mimic the T4 phage tail sheath, cycling between extended and contracted conformations (5–7). Hcp appears to play a multifaceted role in the T6SS (8). Hcp proteins form hexameric rings that coalesce into tubular formations (9). Effector proteins engage with the Hcp ring interiors for targeted secretion. It has been proposed that upon receiving an unknown signal, the VipA/B contraction generates sufficient mechanical force to propel Hcp-tipped effectors into adjacent cells (10, 11).

*V. parahaemolyticus* possesses two T6SS gene clusters. Among them, T6SS2 is ubiquitous, whereas T6SS1 is primarily associated with clinical isolates and has been implicated in adhesion to host cells—a crucial step in establishing infection (12, 13). Beyond mediating bacterial competition, the T6SS of *Vibrio* species, including *V. parahaemolyticus*, can also deliver effector proteins into eukaryotic cells to disrupt host immune responses and promote virulence (14, 15). For instance, effectors from other pathogens can modulate NF-κB signaling to suppress inflammation (16, 17), suggesting that T6SS plays a key role in immune evasion. The enrichment of T6SS1 in clinical isolates implies its contribution to virulence (13); however, some studies have reported that T6SS1 expression is undetectable under laboratory conditions at 37°C, indicating that it may not be involved in mammalian pathogenicity (18).

To investigate whether T6SS1 contributes to the virulence and host interactions of *V. parahaemolyticus*, we constructed single-gene mutants (Δ*vipA1* and Δ*hcp1*) and a double mutant (Δ*vipA1-hcp1*) in the *V. parahaemolyticus* strain SH112. Through proteomic analysis of T6SS1-dependent secretion, phenotypic characterization, and *in vivo* infection assays, we found that VipA1 and Hcp1 are essential for bacterial competition, motility, biofilm formation, and suppression of pro-inflammatory cytokine responses via the NF-κB pathway, collectively enhancing systemic infection. These findings highlight the pivotal role of T6SS1 in immune evasion and its contribution to mammalian virulence, providing new insights into the pathogenesis of *V. parahaemolyticus*.

## RESULTS

### Inactivation of the *vipA1* or/and *hcp1* genes

To investigate the functions of T6SS1, we successfully generated structural protein mutants (Δ*vipA1*, Δ*hcp1*, and Δ*hcp1-vipA1*), as well as complemented strains (CΔ*hcp1* and CΔ*vipA1*). To determine if mutations in *vipA1* and/or *hcp1* had a polar effect on adjacent genes, we used quantitative reverse transcription polymerase chain reaction (qRT-PCR) to assess the transcription levels of *vp1392* (*clpV1*), *vp1394* (*vgrG1*), *vp1401*, and *vp1403* (*vipB1*). The results indicated that deletion of *vipA1* and/or *hcp1* did not impact the expression of upstream and downstream genes within the T6SS1 cluster (data not shown). Furthermore, no significant differences were observed in the generation times and final optical densities of the individual strains when grown in Luria-Bertani (LB) broth supplemented with 3% NaCl (Fig. S1).

### The expression profiles of *V. parahaemolyticus* SH112 strain are affected by VipA1 and/or Hcp1

**Fig 1:**
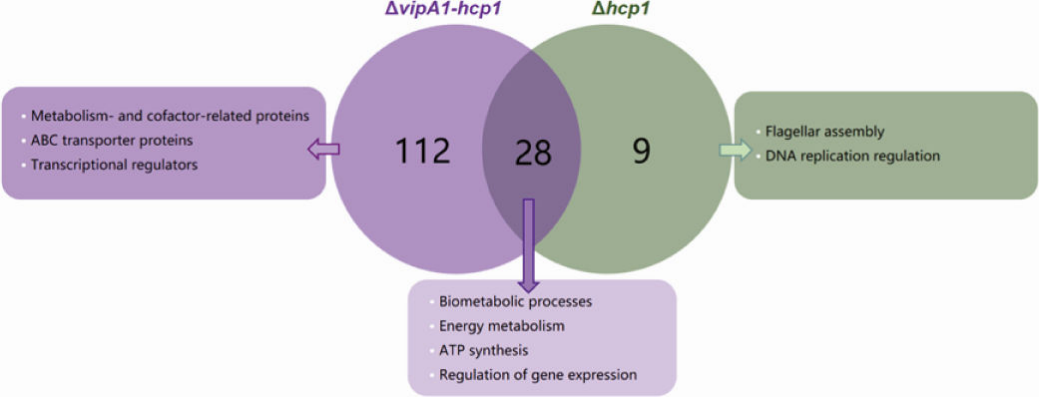
Bioinformatics analysis of VipA1 and Hcp1-dependent secretion in *V. parahaemolyticus* through label-free mass spectrometry. The number of differentially expressed proteins (DEPs) identified in WT and Δ*vipA1-hcp1*/Δ*hcp1* mutant groups, highlighting proteins predominantly expressed in WT strains but absent in mutants.

To verify that mutations in VipA1 and Hcp1 impair the function of T6SS1, we performed comparative proteomic analyses of the Δ*hcp1* and Δ*vipA1-hcp1* mutants vs the wild-type (WT) strain using label-free quantification (LFQ) mass spectrometry. The overall secretome profiles, illustrating T6SS1-dependent secretion patterns, are summarized in Fig. 1. As shown in Table 1, a total of 149 proteins present in the WT supernatant were absent in the mutants. Among them, proteins 1–28 were missing in both Δ*hcp1* and Δ*vipA1-hcp1*, mainly associated with biometabolic processes, energy metabolism and ATP synthesis, and gene expression and RNA modification. Proteins 29–140, which were exclusively undetectable in the Δ*vipA1-hcp1* sample, were primarily involved in metabolic and cofactor processes, ABC transport, and transcriptional regulation. In contrast, proteins 141–149 were absent only in the Δ*hcp1* mutant and were mainly related to flagellar assembly and DNA replication regulation.

**TABLE 1:** The DEPs in Δ*vipA1-hcp1/*Δ*hcp1* and WT groups^*a*^

Number	Protein	Protein name	Gene name	Δ*vipA1-hcp1*	Δ*hcp1*
1	P22099	Anthranilate synthase component 1	*trpE*	*	*
2	P46235	UPF0234 protein VP1617	*VP1617*	*	*
3	P59562	Ferredoxin-type protein	*VPA1486*	*	*
4	Q79YW1	Uncharacterized protein	*VPA1301*	*	*
5	Q87FF4	Putative PmbA-related protein	*VPA0139*	*	*
6	Q87FJ2	Uncharacterized protein	*VPA0106*	*	*
7	Q87FQ1	ATP synthase subunit delta	*atpH*	*	*
8	Q87G17	N5-carboxyaminoimidazole ribonucleotide synthase	*purK*	*	*
9	Q87G28	Gene 3 protein-related protein	*VP3006*	*	*
10	Q87G31	50S ribosomal protein L9	*rplI*	*	*
11	Q87GC0	Nitrogen regulatory IIA protein PtsN	*VP2672*	*	*
12	Q87GL4	BolA/YrbA family protein	*VP2659*	*	*
13	Q87GL7	Aldehyde dehydrogenase	*VP2630*	*	*
14	Q87H05	RNA polymerase sigma factor RpoS	*rpoS*	*	*
15	Q87HF3	ATP-dependent zinc metalloprotease FtsH	*ftsH*	*	*
16	Q87H × 7	Phosphohistidine phosphatase	*VP2205*	*	*
17	Q87I01	Molybdopterin synthase sulfur carrier subunit	*moaD*	*	*
18	Q87I25	Macrodomain Ter protein	*matP*	*	*
19	Q87IG4	Uncharacterized protein	*VP1550*	*	*
20	Q87II4	Putative formate dehydrogenase-specific chaperone	*VP1511*	*	*
21	Q87IK1	Histidine biosynthesis bifunctional protein HisIE	*hisI*	*	*
22	Q87IS8	DNA-binding protein	*VP1133*	*	*
23	Q87J01	Polyphosphate kinase	*ppk*	*	*
24	Q87J30	Peptide chain release factor 2	*VP0512*	*	*
25	Q87J79	3,5-cyclic adenosine monophosphate phosphodiesterase CpdA	*cpdA*	*	*
26	Q87JC6	Uncharacterized protein	*VP0299*	*	*
27	Q87JE9	50S ribosomal protein L16	*rplP*	*	*
28	Q87JL9	Putative regulator	*VP0217*	*	*
29	Q87JM7	Probable lactoylglutathione lyase	*gloA*	*	
30	Q87JS8	Flagellar motor switch protein FliG	*VP2248*	*	
31	Q87JV9	Uncharacterized protein	*VPA1725*	*	
32	Q87JW0	Putative transcriptional regulator	*VPA1687*	*	
33	Q87JZ2	Uncharacterized protein	*VPA1627*	*	
34	Q87K29	Uncharacterized protein	*VPA1500*	*	
35	Q87K59	Uncharacterized protein	*VPA1490*	*	
36	Q87K84	Acyl-CoA thioester hydrolase-related protein	*VPA1397*	*	
37	Q87KA5	Putative glutathione S-transferase	*VPA1298*	*	
38	Q87KB5	Bifunctional NAD(P)H-hydrate repair enzyme	*nnrD*	*	
39	Q87KC6	Cytochrome c554	*VPA1012*	*	
40	Q87KD0	Iron-containing alcohol dehydrogenase	*VPA0829*	*	
41	Q87KE0	Glycine cleavage system protein T2	*VPA0805*	*	
42	Q87KH1	Uncharacterized protein	*VPA0781*	*	
43	Q87KN5	Putative glutathione S-transferase	*VPA0642*	*	
44	Q87KQ8	Putative acyl-CoA dehydrogenase	*VPA0622*	*	
45	Q87KT0	Uncharacterized protein	*VPA0605*	*	
46	Q87KV2	Uncharacterized protein	*VPA0528*	*	
47	Q87L24	Putative DNA-binding stress protein	*VPA0454*	*	
48	Q87L50	Hemin ABC transporter, periplasmic hemin-binding protein HutB	*VPA0423*	*	
49	Q87L61	Periplasmic L-asparaginase II	*VPA0374*	*	
50	Q87L75	Putative translation elongation factor G	*VPA0328*	*	
51	Q87LB0	Uncharacterized protein	*VPA0304*	*	
52	Q87LE0	Putative sugar phosphotransferase component II B	*VPA0230*	*	
53	Q87LE1	Carbonic anhydrase	*VPA0221*	*	
54	Q87LE2	Biosynthetic arginine decarboxylase	*speA*	*	
55	Q87LF2	Putative TldD protein	*VPA0138*	*	
56	Q87LF3	Uncharacterized protein	*VPA0069*	*	
57	Q87LI2	Uncharacterized protein	*VPA0039*	*	
58	Q87LM8	Uncharacterized protein	*VPA0014*	*	
59	Q87LP9	L-threonine dehydratase	*ilvA*	*	
60	Q87LQ8	Uncharacterized protein	*VP3051*	*	
61	Q87LV1	Uncharacterized protein	*VP3047*	*	
62	Q87LY8	Soluble pyridine nucleotide transhydrogenase	*sthA*	*	
63	Q87LZ5	Uncharacterized protein	*VP2918*	*	
64	Q87M01	Bifunctional purine biosynthesis protein PurH	*purH*	*	
65	Q87M39	Fumarate hydratase class II	*fumC*	*	
66	Q87M78	Phosphoribulokinase	*VP2792*	*	
67	Q87MI6	Cystathionine gamma-synthase	*VP2765*	*	
68	Q87MM6	Penicillin-binding protein 1A	*VP2751*	*	
69	Q87MS3	MSHA biogenesis protein MshN	*VP2702*	*	
70	Q87MT1	Putative sigma-54 modulation protein	*VP2671*	*	
71	Q87MV7	RNA polymerase sigma-54 factor	*VP2670*	*	
72	Q87MY3	Putative anti-sigma B factor antagonist	*VP2660*	*	
73	Q87N09	tRNA-modifying protein YgfZ	*VP2583*	*	
74	Q87N88	Cytidine 5'-triphosphate (CTP) synthase	*pyrG*	*	
75	Q87NB7	Pantothenate synthetase	*panC*	*	
76	Q87NC3	Tyrosine—tRNA ligase 2	*tyrS2*	*	
77	Q87ND5	Transcription termination/antitermination protein NusA	*nusA*	*	
78	Q87*N* × 4	Putative pilus assembly transmembrane protein	*VP2419*	*	
79	Q87P91	2,3,4,5-tetrahydropyridine-2,6-dicarboxylate N-succinyltransferase	*dapD*	*	
80	Q87PA9	Succinyl-diaminopimelate desuccinylase	*dapE*	*	
81	Q87PC8	Putative glucose-6-phosphate 1-epimerase	*VP2158*	*	
82	Q87PF2	Putative nitroreductase	*VP2150*	*	
83	Q87PJ1	Aspartate-semialdehyde dehydrogenase	*asd*	*	
84	Q87PJ2	Uridine kinase	*udk*	*	
85	Q87PJ4	Uncharacterized protein	*VP1987*	*	
86	Q87PK6	Ribosomal large subunit pseudouridine synthase B	*rluB*	*	
87	Q87PL8	Transcriptional regulator, LuxR family	*VP1945*	*	
88	Q87PM2	Ubiquinone biosynthesis O-methyltransferase	*ubiG*	*	
89	Q87PQ4	Metal-dependent carboxypeptidase	*VP1744*	*	
90	Q87PY5	Acylphosphatase	*acyP*	*	
91	Q87Q02	Uncharacterized protein	*VP1608*	*	
92	Q87Q75	Iron-sulfur cluster-binding protein	*VP1510*	*	
93	Q87Q92	Uncharacterized protein	*VP1508*	*	
94	Q87QD7	Molybdopterin biosynthesis MoeA protein	*VP1496*	*	
95	Q87QJ9	Uncharacterized protein	*VP1484*	*	
96	Q87QK5	Riboflavin synthase, alpha chain	*VP1480*	*	
97	Q87QK7	Putative anaerobic dimethyl sulfoxide reductase, subunit A	*VP1447*	*	
98	Q87QL6	Putative M20/M25/M40 family peptidase	*VP1365*	*	
99	Q87QL9	Peptidase, M20A family	*VP1348*	*	
100	Q87QU0	Formimidoylglutamase	*hutG*	*	
101	Q87R06	NAD-dependent malic enzyme	*maeA*	*	
102	Q87R29	DNA-binding response regulator	*VP1212*	*	
103	Q87R34	Cysteine—tRNA ligase	*cysS*	*	
104	Q87R43	1-(5-phosphoribosyl)−5-([5-phosphoribosylamino]methylideneamino) imidazole-4-carboxamide isomerase	*hisA*	*	
105	Q87R60	tRNA-specific 2-thiouridylase MnmA	*mnmA*	*	
106	Q87RA3	TolA protein	*VP1059*	*	
107	Q87RB0	Pyruvate formate-lyase-activating enzyme	*VP0992*	*	
108	Q87RB8	UPF0227 protein VP0969	*VP0969*	*	
109	Q87RE4	Hit family protein	*VP0964*	*	
110	Q87RG0	Ferredoxin	*VP0955*	*	
111	Q87RG4	Uncharacterized protein	*VP0938*	*	
112	Q87RG8	Acyl carrier protein	*acpP*	*	
113	Q87RH0	Uncharacterized protein	*VP0887*	*	
114	Q87RH4	Bifunctional protein FolD	*folD*	*	
115	Q87RI8	Zinc ABC transporter, periplasmic zinc-binding protein	*VP0853*	*	
116	Q87RN4	Putative esterase/lipase YbfF	*VP0837*	*	
117	Q87RN8	Glutamine—tRNA ligase	*glnS*	*	
118	Q87RV1	N-acetylglucosamine repressor	*VP0828*	*	
119	Q87R × 5	Asparagine synthetase B, glutamine-hydrolyzing	*VP0826*	*	
120	Q87S24	Adenylate kinase	*adk*	*	
121	Q87S26	Uncharacterized protein	*VP0806*	*	
122	Q87S51	Peptide chain release factor 1	*prfA*	*	
123	Q87SB0	Ribose-phosphate pyrophosphokinase	*prs*	*	
124	Q87SB2	Sigma factor-binding protein Crl	*crl*	*	
125	Q87SB6	Protein GrpE	*grpE*	*	
126	Q87SE4	Chaperone protein HscA homolog	*hscA*	*	
127	Q87SJ5	Iron-binding protein IscA	*VP0598*	*	
128	Q87SS7	Sigma-54-dependent transcriptional regulator	*VP0514*	*	
129	Q87SV8	Flavodoxin	*VP0508*	*	
130	Q87SW6	Uncharacterized protein	*VP0480*	*	
131	Q87S × 3	2-isopropylmalate synthase	*leuA*	*	
132	Q87T06	UDP-N-acetylmuramate-L-alanyl-gamma-D-glutamyl-meso-2,6-diaminoheptandioate ligase	*mpl*	*	
133	Q87T38	Peptide methionine sulfoxide reductase MsrA	*msrA*	*	
134	Q87T53	Putative carbamoylphosphate synthase large subunit, short form	*VP0232*	*	
135	Q87TB6	Transcriptional regulator OmpR	*VP0154*	*	
136	Q87TB7	Putative exported protein	*VP0153*	*	
137	Q87TD8	General secretion pathway protein C	*VP0132*	*	
138	Q87TF4	Coproporphyrinogen-III oxidase	*VP0115*	*	
139	Q87TG4	Uncharacterized protein	*VP0105*	*	
140	Q87TM2	Peptide ABC transporter, ATP-binding protein	*VP0047*	*	
141	Q87HN7	Uncharacterized protein	*VPA0926*		*
142	Q87LD5	UPF0307 protein VP2677	*VP2677*		*
143	Q87QL4	Uncharacterized protein	*VP1135*		*
144	Q87RF9	Negative modulator of initiation of replication	*seqA*		*
145	Q87RK9	Uncharacterized protein	*VP0769*		*
146	Q87RQ7	Endolytic peptidoglycan transglycosylase RlpA	*rlpA*		*
147	Q87SZ4	50S ribosomal protein L15	*rplO*		*
148	Q9 × 9J4	Flagellar P-ring protein 2	*flgI2*		*
149	Q9 × 9J5	Flagellar L-ring protein 1	*flgH1*		*

^*a*^ "*" Represents differentially expressed proteins (DEPs) detected in only WT group in ΔvipA1-hcp 1/Δhcp1 and WT groups.

To gain insights into the functions of these proteins and pinpoint the enriched metabolic and signaling pathways, we conducted Gene Ontology (GO) term enrichment and Kyoto Encyclopedia of Genes and Genomes (KEGG) pathway analyses on the differentially expressed proteins (DEPs). As shown in Fig. S2A and B for the GO term enrichment analysis, biological processes (BPs): the DEPs between WT and ∆*vipA1-hcp1* strains were functionally linked to α-amino acid metabolism, cellular amino acid metabolism, and oxoacid metabolism; the DEPs between WT and ∆*hcp1* were related to DNA metabolism, fatty acid metabolism, and fatty acid biosynthesis. Additionally, the DEPs between WT and ∆*vipA1-hcp1* were primarily associated with molecular functions (MFs) such as small molecule binding, anion binding, and drug binding. In contrast, the DEPs between WT and ∆*hcp1* were mainly related to wide pore channel activity, pore protein activity, and passive transmembrane transporter activity. According to the GO cellular component annotation, the major components of DEPs of WT and ∆*vipA1-hcp1* were cells, cell parts, and membranes, whereas the major components of DEPs between WT and ∆*hcp1* were bacterial-type flagellar basal bodies and distal rod-shaped bacterial-type flagella. Furthermore, we performed KEGG annotation of the DEPs to obtain a comprehensive view of the impact of Hcp1 and VipA1 on the protein profile. As shown in Fig. S2C and D, most DEPs between WT and ∆*vipA1-hcp1* were associated with pathways such as the one-carbon pool by folate, lysine degradation, and histidine metabolism, among others. The DEPs between WT and ∆*hcp1* were associated with beta-alanine metabolism.

### VipA1 and Hcp1 are essential for interbacterial competition in *V. parahaemolyticus*

**Fig 2:**
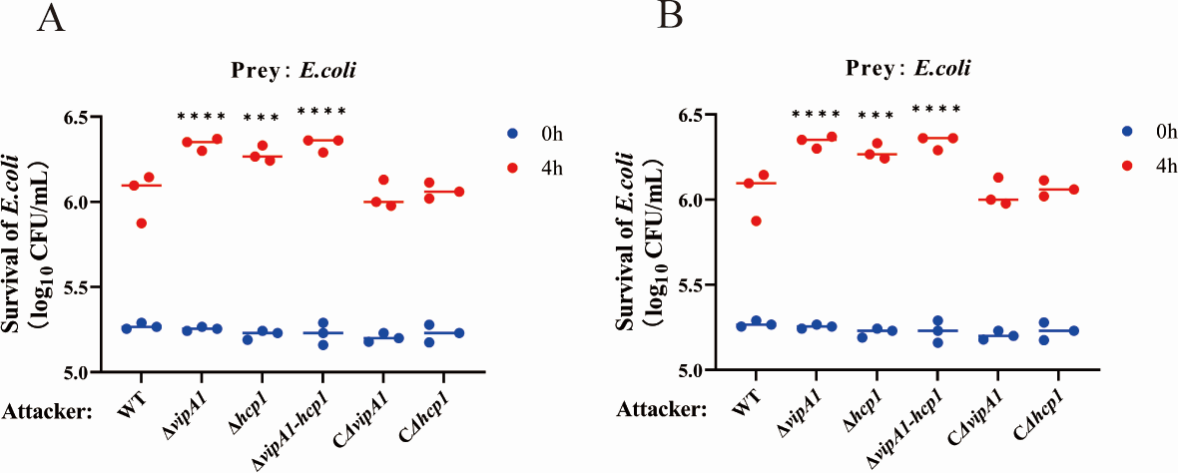
VipA1 and Hcp1 (T6SS1 structural components) mediate interbacterial competition. (**A**) Viability of *E. coli* (prey) and *V. parahaemolyticus* (attacker) before co-culture (0 h) and after 4 h co-incubation at 37°C. (**B**) Survival rates of *V. parahaemolyticus* under identical experimental conditions. Statistical analysis indicated a significant difference (***, *P* < 0.001; ****, *P* < 0.0001).

Bacterial killing assays were conducted to assess whether the deletion of *vipA1* and *hcp1* affects T6SS1-mediated antibacterial competition. As shown in Fig. 2A, following 4 h incubation at 37°C, prey (*Escherichia coli*) viability was significantly elevated in co-cultures with Δ*vipA1* (53.9%), Δ*hcp1* (33.8%), and Δ*vipA1-hcp1* (52.6%) mutants relative to the WT strain (*P* < 0.001; *P* < 0.0001). Complementation with CΔ*vipA1* and CΔ*hcp1* rescued bacterial killing capacity to WT levels. Furthermore, the survival rates of all mutant strains showed no significant difference from the WT (Fig. 2B), ruling out potential confounding effects due to impaired fitness of the mutants. Collectively, these data demonstrate that VipA1 and Hcp1 coordinately regulate T6SS1-mediated antibacterial activity through synergistic interactions.

### Differential effects of VipA1 and Hcp1 on biofilm formation

**Fig 3:**
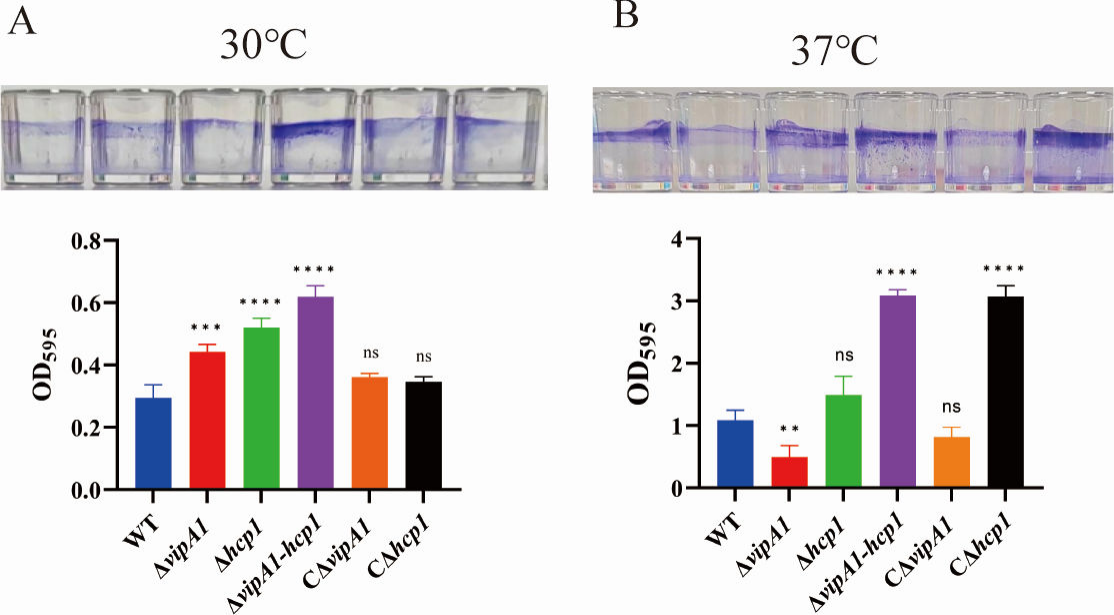
VipA1 and Hcp1 modulate biofilm formation. (**A and B**) Static biofilm formation by WT, mutant, and complemented strains at 30°C and 37°C, visualized and quantified via ethanol solubilization. The asterisks indicate significant differences. Statistical analysis indicated a significant difference (ns: not significant; *, *P* < 0.05; ***, *P* < 0.001; ****, *P* < 0.0001).

Biofilm formation by each strain was assessed at 30°C and 37°C under static culture conditions. As shown in Fig. 3A, the ∆*vipA1*, ∆*hcp1*, and ∆*vipA1-hcp1* mutants exhibited 1.5-, 1.8-, and 2.1-fold increases in biofilm biomass at 30°C, respectively, compared with the WT (*P* < 0.001; *P* < 0.0001). Genetic complementation restored biofilm formation to WT levels. At 37°C, ∆*vipA1* showed reduced biofilm formation (0.5-fold vs WT; *P* < 0.05, Fig. 3B). Intriguingly, ∆*hcp1* displayed 1.4-fold higher biofilm biomass than WT (*P* > 0.05), the ∆*vipA1-hcp1* double mutant demonstrated 2.8-fold enhancement (*P* < 0.0001). Both CΔ*vipA1* and CΔ*hcp1* showed partial restoration of biofilm formation to the level of the WT strain. Comparative analysis revealed that ∆*vipA1-hcp1* produced significantly more biofilm than single mutants (∆*vipA1* and ∆*hcp1*) at both 30°C and 37°C (*P* < 0.05; *P* < 0.0001). Strikingly, ∆*vipA1* displayed divergent phenotypes: enhanced biofilm formation at 30°C vs suppressed formation at 37°C, revealing temperature-dependent regulation by VipA1. The remarkable increase in biofilm formation upon co-deletion of *vipA1* and *hcp1* indicates a synergistic interaction between these two genes.

### VipA1 and Hcp1 are indispensable for the motility of SH112

**Fig 4:**
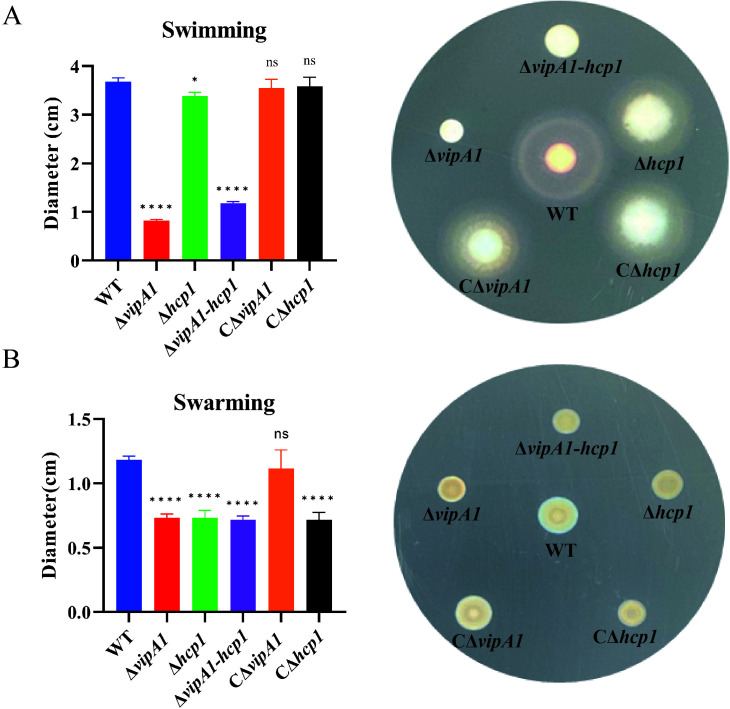
VipA1 and Hcp1 regulate motility phenotypes in *V. parahaemolyticus*. (**A**) Swimming motility assay: bacterial migration on 0.3% agar LB plates with 1% NaCl at 37°C for 4 h. (**B**) Swarming motility assay: bacterial migration on 1.5% agar brain heart infusion plates with 2% NaCl at 30°C for 15–20 h. Statistical analysis indicated a significant difference (ns: not significant; *, *P* < 0.05; ****, *P* < 0.0001).

To assess the contribution of *vipA1* and *hcp1* to bacterial motility, we conducted quantitative dynamic monitoring of bacterial motility. As shown in Fig. 4A, T6SS1 mutants (Δ*vipA1*, Δ*hcp1*, and Δ*vipA1-hcp1*) exhibited significantly reduced swimming motility, with diameters decreased to 22.3%, 86.5%, and 30.7% of WT levels, respectively (*P* < 0.05; *P* < 0.0001). Complementation fully restored swimming motility to WT capacity. Figure 4B demonstrates that swarming motility diameters of Δ*vipA1*, Δ*hcp1*, and Δ*vipA1-hcp1* were reduced to 40.0%, 39.0%, and 35.5% of WT, respectively (*P* < 0.0001). While CΔ*hcp1* exhibited restored swarming motility comparable to WT levels, CΔ*vipA1* showed limited phenotypic recovery under the same conditions. Collectively, T6SS1-mediated inactivation of Hcp1 and VipA1 severely impaired both swimming and swarming motility, demonstrating their essential roles in *V. parahaemolyticus* locomotion regulation.

### Deletion of *vipA1* and *hcp1* diminishes bacterial pathogenicity

**Fig 5:**
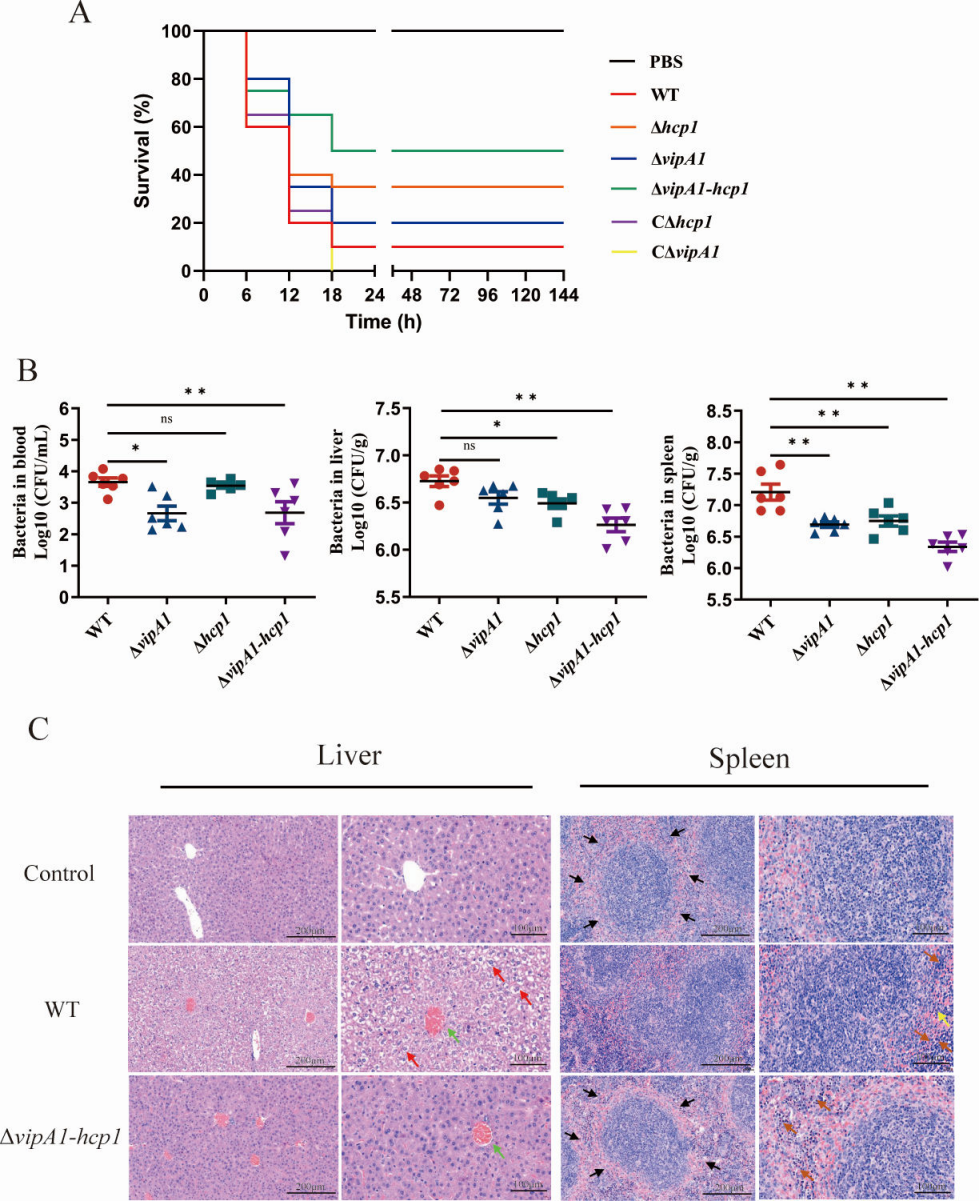
VipA1 and Hcp1 are required for *V. parahaemolyticus* virulence *in vivo*. (**A**) Survival curves of Institute for Cancer Research (ICR) mice (*n* = 12/group) infected intraperitoneally with 10⁷ CFU WT/mutants; PBS controls included. (**B**) Bacterial loads in blood/liver/spleen (*n* = 6) at 20 h post-infection. *, *P* < 0.05; **, *P* < 0.01 (Mann-Whitney *U*-test). (**C**) Hematoxylin and eosins tained liver/spleen sections: uninfected controls (intact hepatic cords/sinusoids); WT-infected livers (red arrows = edema/degeneration; green arrows = thrombosis); WT-infected spleens (black arrows = disrupted white pulp; orange arrows = lymphocyte accumulation; yellow arrows = neutrophils). Scale bars: ×200 and ×400. Statistical analysis indicated a significant difference (ns: not significant; *, *P* < 0.05; **, *P* < 0.01).

Mice infected with the WT strain exhibited a survival rate of 8.3% (1/12), whereas those challenged with the Δ*vipA1*, Δ*hcp1*, and Δ*vipA1-hcp1* mutants showed increased survival rates of 16.7% (2/12), 33.3% (4/12), and 50% (6/12), respectively. Genetic complementation with CΔ*hcp1* fully restored survival to WT levels (8.3%, 1/12), while CΔ*vipA1* displayed partial restoration of survival, albeit at a rate lower than WT. Phosphate-buffered saline (PBS)-treated control mice displayed 100% survival (12/12; Fig. 5A). Post-dissection analysis revealed that *vipA1* and/or *hcp1* deletion significantly impaired bacterial colonization in the blood, liver, and spleen *in vivo* (*P* < 0.05 ; *P* < 0.01; Fig. 5B). The Δ*vipA1-hcp1* double mutant exhibited significantly more severe colonization defects compared to the WT strain, with 15-, 3-, and 10-fold reductions in bacterial loads in the blood, liver, and spleen, respectively (*P* < 0.01). CΔ*vipA1* and CΔ*hcp1* partially restored colonization capacity to the WT level (data not shown). *In vivo* histopathological examination (Fig. 5C) showed that WT infection induced severe liver and spleen pathology, characterized by cellular edema, fibrin deposition, inflammatory cell infiltration, and vesicular degeneration. In contrast, Δ*vipA1-hcp1*-infected mice showed no detectable pathological alterations. These findings establish that VipA1 and Hcp1 critically govern *V. parahaemolyticus* colonization and survival *in vivo*, thereby directly validating their indispensable contributions to the pathogen’s virulence.

### VipA1 and Hcp1 affect cytotoxicity in host cells

**Fig 6:**
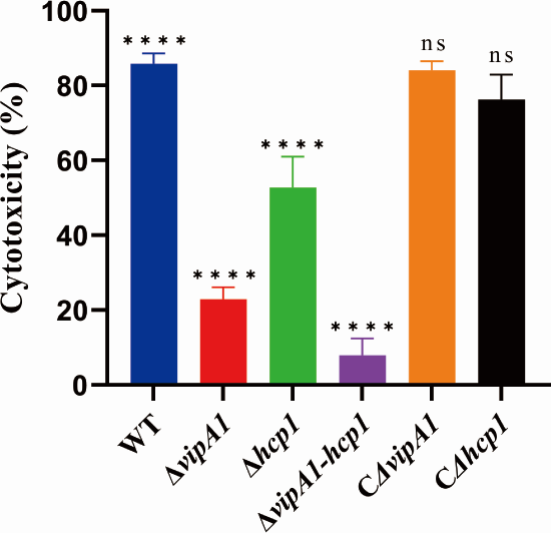
VipA1 and Hcp1 drive host cell cytotoxicity. Lactate dehydrogenase (LDH) release assay of HeLa cells infected with WT/mutant/complemented strains (Multiplicity of infection [MOI] = 100, 1 h). Statistical analysis indicated a significant difference (ns: not significant; ****, *P* < 0.0001).

Cytotoxicity assays showed that infection with Δ*vipA1*, Δ*hcp1*, and Δ*vipA1-hcp1* mutants reduced HeLa cell lysis rates by 62.8%, 32.8%, and 77.6%, respectively, compared to the WT strain (*P* < 0.0001; Fig. 6). Genetic complementation fully restored cytotoxicity to WT levels, confirming that VipA1 and Hcp1 are essential for mediating HeLa cell death *in vitro*. The Δ*vipA1-hcp1* double mutant displayed significantly weaker cytotoxicity than either Δ*vipA1* (*P* < 0.0001) or *Δhcp1* (*P* < 0.05) single mutants, demonstrating a synergistic interaction between VipA1 and Hcp1 in driving host cell lysis.

### VipA1 and Hcp1 synergistically suppress pro-inflammatory cytokine transcription

**Fig 7:**
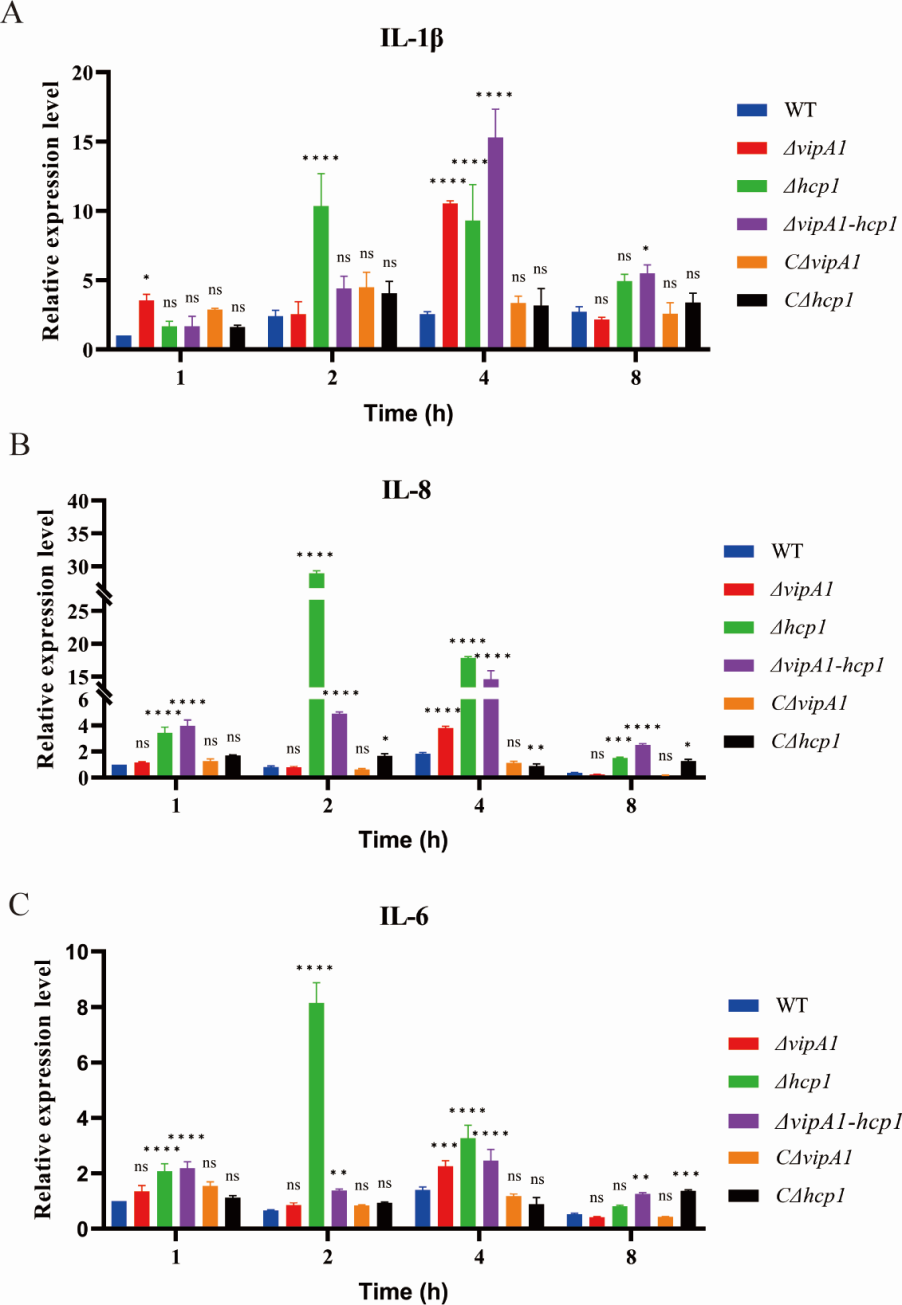
VipA1 and Hcp1 suppress pro-inflammatory cytokine transcription. qPCR analysis of (**A**) IL-1β, (**B**) IL-8, and (**C**) IL-6 mRNA levels post-infection (MOI = 10). Statistical analysis indicated a significant difference (ns: not significant; *, *P* < 0.05; **, *P* < 0.01; ***, *P* < 0.001; ****, *P* < 0.0001).

Compared to the WT strain, the Δ*vipA1* mutant significantly elevated IL-1β mRNA levels at 1 h (*P <* 0.05*)* and 4 h *(P* < 0.0001) post-infection and increased both IL-8 and IL-6 mRNA levels at 4 h (*P* < 0.001; Fig. 7). The Δ*hcp1* mutant induced cytokine mRNA upregulation at most time points (*P* < 0.0001; *P* < 0.001; *P* < 0.01). The Δ*vipA1-hcp1* double mutant showed heightened IL-1β levels at 4 h (*P* < 0.0001) and 8 h (*P* < 0.01), with sustained IL-8 and IL-6 overexpression across all infection stages (*P* < 0.0001; *P* < 0.01). These results indicate that VipA1 and Hcp1 suppress proinflammatory cytokine transcription to promote *V. parahaemolyticus* infection *in vivo*.

### VipA1 and Hcp1 synergistically inhibit IκBα degradation and impede p65 nuclear translocation

**Fig 8:**
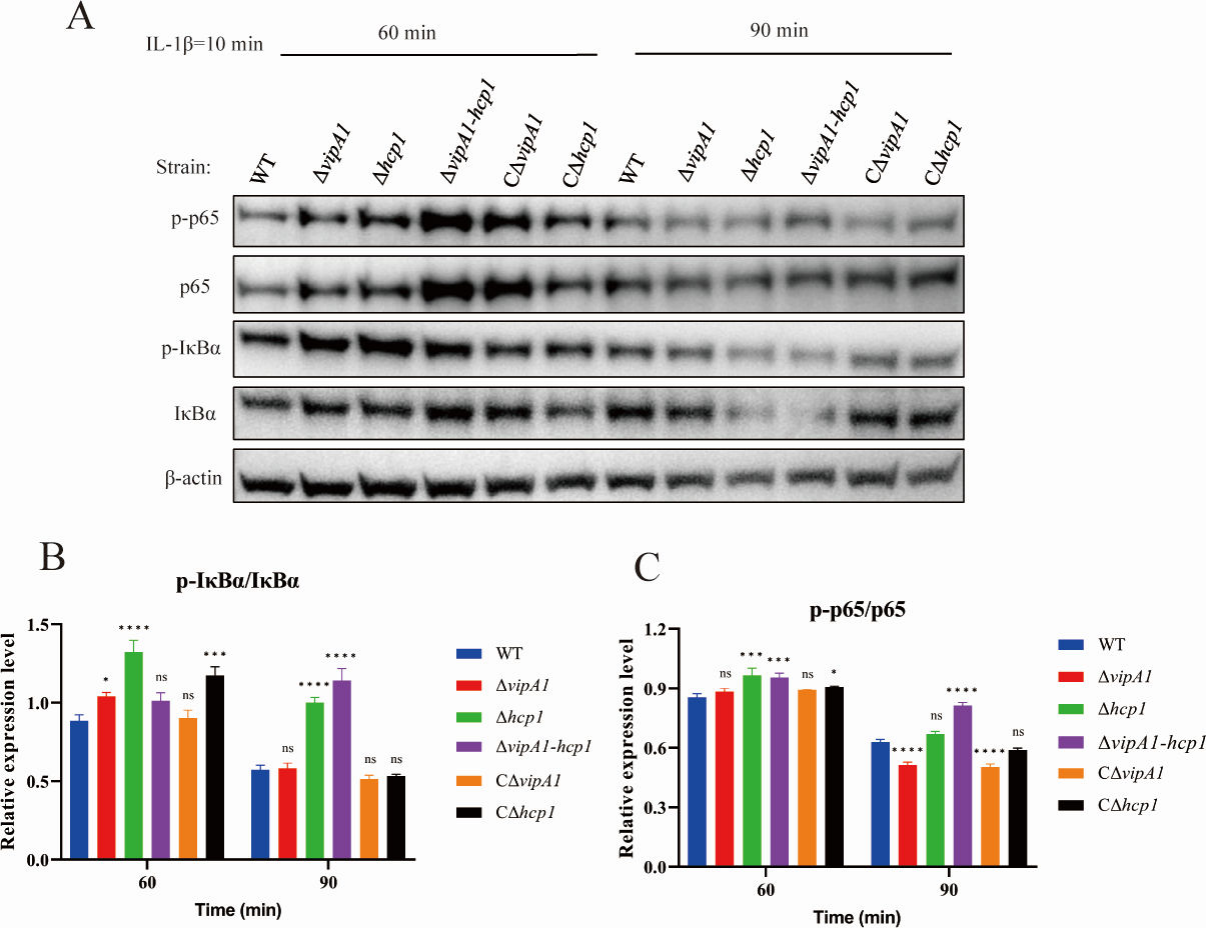
VipA1 and Hcp1 attenuate NF-κB signaling in infected HeLa cells. (**A**) Total and phosphorylated forms of IκBα and p65 were evaluated via immunoblotting at 60 and 90 min post-IL-1β stimulation (10 ng/mL). β-Actin served as a loading control. (**B and C**) Densitometric analysis of immunoblots from three independent experiments (mean ± SD) shows the level of significance. Statistical analysis indicated a significant difference (ns: not significant; *, *P* < 0.05; ***, *P* < 0.001; ****, *P* < 0.0001).

To investigate whether VipA1 and Hcp1 enhance *V. parahaemolyticus* pathogenicity through NF-κB pathway modulation, we analyzed IκBα degradation and p65 phosphorylation kinetics. HeLa cells were infected with WT, ∆*vipA1*, ∆*hcp1*, ∆*vipA1-hcp1*, C∆*vipA1*, and C∆*hcp1* strains for 1 h and were stimulated with IL-1β. Western blot analysis showed that at 60 min post-stimulation, p65 phosphorylation levels were significantly increased in ∆*hcp1*- and ∆*vipA1-hcp1*-infected cells compared with WT-infected cells (*P* < 0.01; *P* < 0.001; Fig. 8B). By 90 min, p65 phosphorylation of ∆*vipA1-hcp1*-infected cells exceeded WT levels (*P* < 0.0001). Compared to WT, Δ*vipA1*- and Δ*hcp1*-infected cells showed elevated IκBα phosphorylation at 60 min (*P* < 0.05; *P* < 0.0001; Fig. 8C). Furthermore, Δ*hcp1*- and Δ*vipA1-hcp1*-infected cells displayed accelerated IκBα degradation at 90 min (*P* < 0.0001), but phosphorylation levels of CΔ*hcp1* failed to revert back to the WT level. Both the CΔ*vipA1* and CΔ*hcp1* complemented strains exhibited phenotypes restored to WT levels, confirming the functional recovery of VipA1 and Hcp1 in *V. parahaemolyticus*.

**Fig 9:**
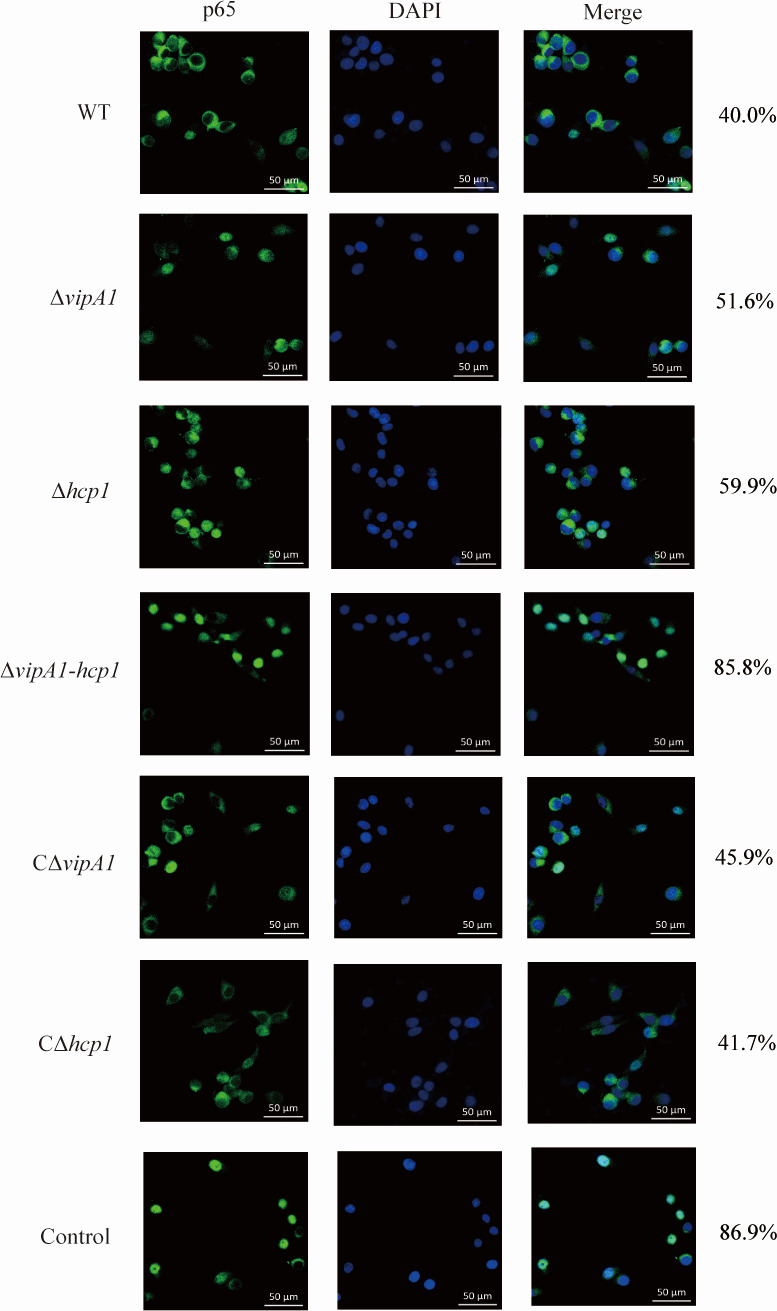
VipA1 and Hcp1 impair NF-κB p65 nuclear translocation. Immunofluorescence microscopy of p65 (green)/diamidino-2-phenylindole (DAPI) (blue) 60 min post-IL-1β stimulation. Nuclear p65 quantification: WT = 40.0%; Δ*vipA1* = 51.6%; Δ*hcp1* = 59.9%; Δ*vipA1-hcp1* = 85.8%. Scale bar: 50 µm.

Confocal immunofluorescence microscopy confirmed enhanced p65 nuclear translocation in Δ*vipA1*- (51.6%), Δ*hcp1*- (59.9%), and Δ*vipA1-hcp1*-infected cells (85.8%) compared to WT (40.0%) at 60 min post-stimulation (Fig. 9). Strikingly, p65 nuclear entry in Δ*vipA1-hcp1*-infected cells (85.8%) matched uninfected controls (86.9%). These results demonstrate that T6SS1 attenuates NF-κB activation by delaying IκBα degradation and suppressing p65 nuclear translocation, directly linking T6SS1-mediated immune evasion to *V. parahaemolyticus* pathogenicity.

## DISCUSSION

T6SS is a versatile nanoweapon employed by gram-negative bacteria to mediate interbacterial competition, biofilm dynamics, and host–pathogen interactions (19, 20). Although T6SS1 has been recognized as a key virulence determinant in *V. parahaemolyticus*, its role in mammalian infection and the underlying mechanisms remain unclear. In this study, by systematically characterizing single (Δ*vipA1* and Δ*hcp1*) and double (Δ*vipA1-hcp1*) mutants of conserved structural components, we elucidate how T6SS1 contributes to the virulence of *V. parahaemolyticus*.

To systematically investigate T6SS1’s pleiotropic effects, we employed comparative proteomic analysis coupled with LFQ to characterize its secretome, with the results summarized in Table 1. Our analysis revealed a complex secretion profile where 149 proteins showed differential abundance between WT and mutant strains (Δ*hcp1* and Δ*vipA1-hcp1*): 37 proteins (No. 1–28 and 141–149) were absent in Δ*hcp1* mutant, while 140 proteins (No. 1–140) showed secretion defects in Δ*vipA1-hcp1* mutant. Notably, the 28 core proteins (Table 1; No. 1–28) requiring both VipA1 and Hcp1 for secretion were functionally enriched in essential BPs, including biometabolism (TrpE, PurK, and HisI), energy/ATP synthesis (AtpH and MoaD), gene expression regulation (RpoS and CpdA), and translation/RNA modification. Notably, GO and KEGG analyses demonstrated distinct functional clustering of differentially secreted proteins: Δ*vipA1-hcp1* mutants showed predominant defects in α-amino acid and oxoacid metabolism pathways, while Δ*hcp1* specifically affected DNA and fatty acid metabolism. This functional divergence was further reflected in MF annotations, with Δ*vipA1-hcp1* secretion-deficient proteins enriched in small molecule/drug binding activities, whereas Δ*hcp1* secretion-deficient proteins (No.141–149) were primarily involved in flagellar assembly and transmembrane transport, consistent with observed motility defects. Particularly noteworthy was the identification of 121 differentially secreted proteins (No. 29–140 in Δ*vipA1-hcp1*; No. 141–149 in Δ*hcp1*) that may mediate VipA1-Hcp1 synergy, including key virulence factors like GrpE, OmpR, and ADK (21–23). Mechanistically, T6SS1 inactivation disrupted multiple cellular subsystems, including (Fig. 1) (i) membrane transport (ABC transporters, outer membrane proteins); (ii) energy metabolism (ATP synthases, oxidative phosphorylation); and (iii) structural components (flagellar basal bodies). These comprehensive proteomic findings establish T6SS1 as a central regulator of *V. parahaemolyticus* physiology, coordinating diverse metabolic and virulence pathways through VipA1-Hcp1-mediated secretion, and provide a molecular framework for future investigations into T6SS1’s role in bacterial pathogenesis.

T6SS represents a sophisticated protein secretion apparatus that orchestrates both interbacterial competition and eukaryotic cell pathogenesis. Our comprehensive analysis of VipA1 and Hcp1 in *V. parahaemolyticus* reveals their essential and coordinated roles in T6SS1-mediated functions. The T6SS has been shown to play a crucial role in mediating bacterial interactions and competitive behaviors across various species, including *V. parahaemolyticus* (24) and *Pseudomonas aeruginosa* (25). The antibacterial competition assays demonstrate that while both structural components contribute to bacterial killing, VipA1 plays a more dominant role in effector secretion, as evidenced by the more severe killing defect in Δ*vipA1* (53.9% reduction) compared to Δ*hcp1* (33.9% reduction; *P* < 0.0001, Fig. 2A). This functional hierarchy is maintained in the Δ*vipA1-hcp1* double mutant, with complementation analysis confirming the specificity of these effects. Previous studies have identified the involvement of Hcp1 in bacterial killing activity in *V. parahaemolyticus* (18), and VipA has also been demonstrated to participate in bactericidal functions in other bacterial species (26). Our current study further reveals that VipA1 serves as the primary determinant of T6SS1-mediated antibacterial activity, while Hcp1 plays an auxiliary yet indispensable role in maintaining the structural integrity required for effector secretion.

Members of the *Vibrio* spp. exhibit robust biofilm-forming capabilities, which may play critical roles in environmental persistence and bacterial pathogenesis (27). The marked enhancement of biofilm formation in ∆*vipA1*, ∆*hcp1*, and ∆*vipA1-hcp1* mutants at 30°C suggests that both VipA1 and Hcp1 function as negative regulators of biofilm production. The synergistic effect observed in the double mutant (2.1-fold increase vs WT) implies that these two components may act through distinct yet complementary pathways to suppress biofilm accumulation. The proteomic data of this study show that VipA1 and Hcp1 coordinate the secretion of structural and metabolic proteins (e.g., FtsH, MatP, and AtpH), which are capable of inhibiting the formation of biological periplasm (28–31). Intriguingly, the temperature-dependent phenotypic divergence of ∆*vipA1*-enhanced biofilm formation at 30°C but reduced formation at 37°C highlights a dual regulatory role for VipA1 in response to environmental cues. This phenomenon could be linked to its interaction with temperature-sensitive substrates, such as RpoS or OmpR, which are known to modulate bacterial stress responses and virulence gene expression (32, 33). The pronounced biofilm hyperproduction in ∆*vipA1-hcp1* at 37°C (2.8-fold vs WT) further indicates that Hcp1 may partially compensate for VipA1’s regulatory functions under thermal stress, possibly through its involvement in energy metabolism (e.g., ATP synthases) or membrane integrity maintenance (e.g., ABC transporters), as identified in our proteomic analysis (Table 1). Contrary to the typical association between biofilm formation and enhanced virulence, our Δ*vipA1-hcp1* mutant displays increased biofilm production coupled with significantly attenuated pathogenicity, indicating that VipA1 and Hcp1 regulate *V. parahaemolyticus* virulence through biofilm-independent pathways. This dissociation between biofilm formation and virulence phenotypes underscores the complexity of T6SS1-mediated pathogenicity regulation in *V. parahaemolyticus*.

The severe motility defects observed in the mutants are consistent with a disruption of T6SS1 function. This is mechanistically supported by our proteomic data, which showed deficient secretion of flagellar assembly proteins (FlgI2 and FlgH1) in the absence of a functional T6SS1, consistent with previous reports linking T6SS components to motility defects in *V. cholerae* (*icmF*), Avian pathogenic *E. coli* (*clpV*), and *Citrobacter freundii* (*hcp2*) (34–36). Notably, the observed motility impairment may be partially attributed to the absence of secreted regulators, as RpoS deficiency has been shown to upregulate *Vibrio* motility proteins (29, 37), while FtsH depletion correlates with reduced swimming capacity and virulence (38, 39). Based on these observations, we postulate that mutations in the *vipA1* and *hcp1* genes may disrupt bacterial flagellum assembly, ultimately leading to impaired motility. These findings significantly expand our understanding of how T6SS1 components orchestrate motility in *V. parahaemolyticus*.

Our comprehensive analyses demonstrate that VipA1- and Hcp1-dependent T6SS1 functions as a critical virulence determinant in *V. parahaemolyticus*. The Δ*vipA1-hcp1* double mutant showed a decrease in mortality from 91.7% (WT) to 50% (Fig. 5A) and the most pronounced decrease in organ colonization (Fig. 5B), and the dramatic attenuation observed in the mutant strains provides compelling *in vivo* evidence for an important role of VipA1 and Hcp1. The complete absence of pathological alterations in Δ*vipA1-hcp1*-infected mice (Fig. 5C), coupled with the 77.6% reduction in HeLa cell lysis (*P* < 0.0001, Fig. 6), strongly suggests that these components function synergistically to mediate host damage. This synergistic relationship is further supported by the more severe virulence attenuation in the double mutant compared to single deletions, mirroring findings in *V. cholerae* (40, 41) and *Salmonella typhimurium* (42). It is noteworthy that in *E. coli*, the structural protein Hcp has been shown to directly interact with host cells and promote colonization (43). Therefore, we do not exclude the possibility that the reduced virulence observed in the Δ*hcp1* mutant results from both impaired T6SS1 function and the absence of Hcp-mediated host interactions.

Mechanistically, our proteomic data (Table 1) reveal that T6SS1 deficiency disrupts multiple virulence-associated pathways: (i) iron acquisition through heme ABC transporter and heme binding protein (HutB) dysfunction (44), (ii) cyclic adenosine monophosphate (cAMP)-mediated signaling via the 3,5-cyclic adenosine monophosphate phosphodiesterase (CpdA) alteration (45), and (iii) stress adaptation through BolA/YrbA family protein dysregulation (46, 47). The decrease in heme utilization and consequent iron limitation likely contributes significantly to the observed attenuation (44), while the disruption of cAMP homeostasis through CpdA may impair host cell invasion (45). These multifaceted effects collectively explain the profound virulence reduction in T6SS1 mutants. These findings not only confirm the conserved pathogenic roles of T6SS components across species (48–50) but also provide novel insights into the specific mechanisms by which VipA1 and Hcp1 coordinately regulate *V. parahaemolyticus* virulence through both direct cytotoxic effects and indirect modulation of essential physiological processes.

We demonstrate that T6SS1 targets the NF-κB pathway in host cells. Several effectors have been found to influence the activity of key signaling pathways, such as NF-κB and MAPK signaling, leading to decreased production of pro-inflammatory cytokines and/or suppression of host inflammation. For instance, the T3SS1 effector VopZ has been shown to inhibit the activation of both MAPK and NF-κB signaling pathways (51). This indicates that *V. parahaemolyticus* uses effector proteins to suppress host inflammation, enhancing intracellular survival. However, the exact mechanism by which T6SS1 influences the host innate immune response remains unclear. Our data demonstrate that a functional T6SS1 is required to suppress the NF-κB pathway in host cells. Disruption of T6SS1 through deletion of its essential structural components, VipA1 and Hcp1, abrogates this immunosuppressive capability. This is evidenced by the failure to prevent IκBα degradation and p65 phosphorylation in host cells infected with the mutant strains, culminating in the upregulated transcription of pro-inflammatory cytokines (IL-1β, IL-8, and IL-6). Consequently, the robust host immune response likely contributes to the accelerated clearance of T6SS1-deficient mutants, explaining their attenuated virulence. In conclusion, our study demonstrates that the structural integrity of T6SS1, maintained by VipA1 and Hcp1, is a prerequisite for its role as a master virulence regulator in *V. parahaemolyticus*. Recent research has demonstrated that effector proteins secreted by the T6SS can interfere with host signaling pathways, disrupting host immune responses. For instance, TssJ-3, an outer membrane lipoprotein of the *Burkholderia cepacia* T6SS, has been observed to translocate to target cells, triggering an innate immune response in the host that favors bacterial survival and virulence within the host cells (20). *Klebsiella pneumoniae* VgrG4 suppresses NF-κB via NLRX1 interaction, reducing inflammatory responses (17). These discoveries suggest that the T6SS provides favorable conditions for bacterial survival, colonization, and infection. By mediating the secretion of a vast repertoire of effectors, T6SS1 coordinates interbacterial competition, environmental adaptation, and, crucially, the suppression of host innate immunity. The synergistic severity of the double-mutant phenotypes highlights the interdependence of these core components. Therefore, targeting the T6SS1 apparatus or its essential structural elements like VipA1 and Hcp1 presents a promising strategy for antivirulence therapy.

**Fig 10:**
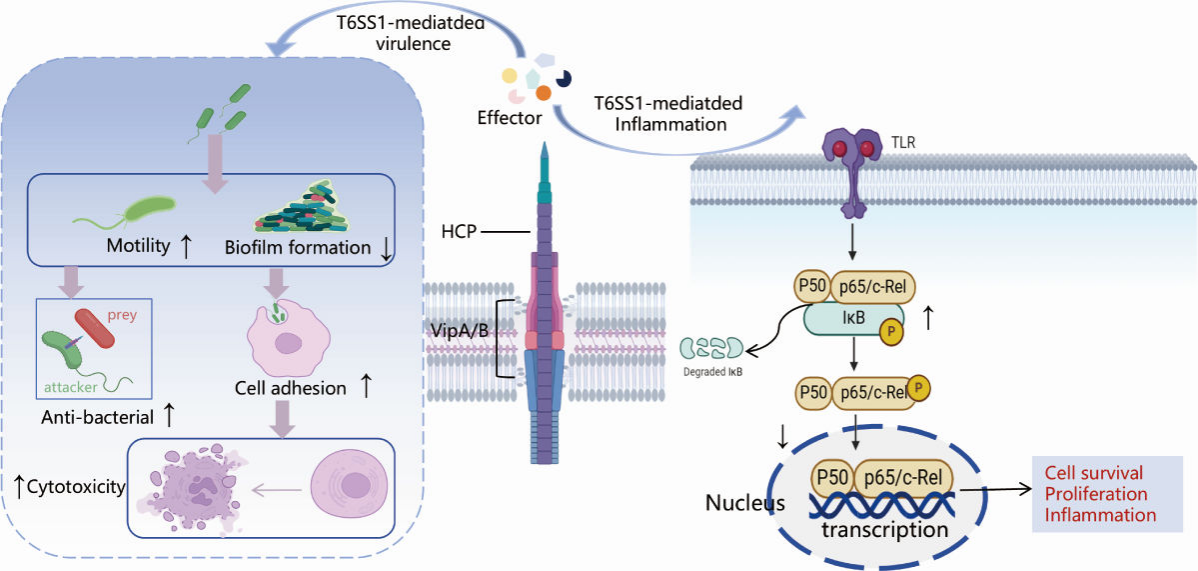
Proposed model of T6SS1 in virulence regulation and immune evasion. Integrated functional analyses reveal that T6SS1 regulates multiple virulence-related processes: environmental adaptation through enhanced interbacterial competition, biofilm formation, and motility; as well as host interactions via increased cytotoxicity and suppression of NF-κB activation and pro-inflammatory cytokine production. Arrows (↑/↓) denote observed enhancement/inhibition of processes.

This study (summarized in Fig. 10) provides comprehensive insights into the coordinated functions of VipA1 and Hcp1 in mediating *V. parahaemolyticus* pathogenesis through T6SS1. Our integrated analyses demonstrate that these structural components orchestrate multiple virulence determinants: they regulate environmental adaptation via interbacterial competitiveness, biofilm formation, and motility modulation, and mediate host interactions by increasing cytotoxicity while suppressing NF-κB activation and cytokine production. Proteomic profiling established direct correlations between VipA1/Hcp1-dependent secretory profiles and functional outcomes, revealing their dual roles in both structural integrity and pathogenic regulation. Importantly, we elucidate how T6SS1 facilitates infection by simultaneously promoting cytotoxicity and immune evasion through precise manipulation of host signaling pathways. These findings not only advance our understanding of T6SS1-mediated pathogenesis but also identify VipA1 and Hcp1 as promising targets for therapeutic intervention, with future studies poised to exploit these components and their associated effectors for vaccine development against *V. parahaemolyticus* infections.

## MATERIALS AND METHODS

### Bacterial strains, plasmids, and growth conditions

The bacterial strains and plasmids used in this study are listed in Table 2. The *V. parahaemolyticus* SH112 strain (GenBank: JACYGZ000000000.1), isolated from a clinical specimen in Shanghai and deposited in the China General Microbiological Culture Collection Center with the accession number CGMCC 1.90013, was used as the WT strain for mutant construction. For cloning, strain CC118λpir facilitated plasmid transfer into *V. parahaemolyticus. E. coli* strains were cultivated at 37°C in LB broth. *V. parahaemolyticus* strains were grown at 37°C in 3% NaCl-LB broth. For screening mutant strains, thiosulfate-citrate-bile salts-sucrose agar supplemented with 10 µg/mL chloramphenicol was used.

**TABLE 2:** Strains and plasmids used in this study

Strain or plasmid	Description	Reference or source
*E. coli* strain		This study
CC118λpir	Λpir lysogen of CC118 Δ (*ara-leu*) *ara*D *ΔlacX74 galE galK phoA20 thi-1 rpsE rpoB argE(*Am*) recA1*	52
*V. parahamolyticus* strain		This study
SH112	WT, Cm^s^	This study
Δ*vipA1*	Mutation in *vipA1* gene of Strain SH112	This study
Δ*hcp1*	Mutation in *hcp1* gene of Strain SH112	This study
Δ*vipA1-hcp1*	Mutation in *vipA1* and *hcp*1 of Strain SH112	This study
CΔ*vipA1*	Δ*vipA1*with plasmid pMMB207- vipA1	This study
CΔ*hcp1*	Δ*hcp1* with plasmid pMMB207- *hcp1*	This study
pMD18T	A clone vector, Amp^r^, lacZ	Takara
pYAK1	A suicide vector with ori R6K sacB; Cm ^r^	52
pMMB207	RSF1010 derivative, *Inc*Q *lac*I q Cm^r^ P*tac ori*T	52
pET28a	Kan, F1 origin, His tag	Novagen

### Inactivation of the *vipA1* and *hcp1* genes

Mutants were constructed through homologous recombination (53). Primer pairs A/B and C/D (Table S1) amplified upstream and downstream fragments of the target genes from SH112 chromosomal DNA. These fragments were fused using primer pair A/D to create flanking regions. The suicide plasmid pYAK1 was ligated with these PCR products to generate recombinant plasmids pYAK1-*vipA1/hcp1* (52). These plasmids were transformed into *E. coli* CC118λpir and subsequently conjugated into the SH112 strain, yielding single mutants ∆*vipA1* and ∆*hcp1*. The ∆*vipA1-hcp1* double mutant was constructed analogously and validated by PCR and sequencing.

The full-length open reading frames of *vipA1* and *hcp1* were amplified from the SH112 genome using primers pMMB-*vipA1*/*hcp1*-F/R (Table S1) and cloned into plasmid pMMB207 to generate pMMB207-*vipA1* and pMMB207-*hcp1*. These plasmids were introduced into Δ*vipA1* and Δ*hcp1* mutants, yielding complementary strains designated C∆*vipA1* and C∆*hcp1*, respectively.

### Sample preparation and LFQ mass spectrometry

The WT, ∆*hcp1,* and ∆*vipA1-hcp1* strains were incubated in 50 mL of Dulbecco’s modified Eagle’s medium (DMEM) at 30°C under vigorous agitation for 6 h, followed by centrifugation at 5,000 × *g* for 30 min at 4°C. Secreted proteins in the culture supernatant were analyzed via a customized LFQ mass spectrometry platform (APTBIO, Shanghai, China). Briefly, 40 mL of supernatant was freeze-dried using liquid nitrogen and lysed in 1 mL of SDS DTT Tris buffer (4% SDS [wt/vol], 100 mM Tris/HCl pH 7.6, 0.1 M DTT). Protein concentrations were quantified using the bicinchoninic acid assay. Proteins were digested with trypsin to generate peptide mixtures, which were desalted using C18 cartridges. Peptides were separated on an EASY-nLC1200 HPLC system with a gradient of 0.1% formic acid (FA) in water and 84% acetonitrile (0.1% FA) at 300 nL/min. Peptide identification and quantification were performed using Q-Exactive mass spectrometry. DEPs were defined by thresholds of *P* < 0.05 and fold changes >2.0 or <0.5. GO and KEGG pathway analyses were executed using Fisher’s exact test.

### Bacterial killing assays

Bacterial competition assays were conducted as previously described (18). Briefly, both the attacker (*V. parahaemolyticus*) and prey (*E. coli*) were uniformly incubated at 37°C, normalized to an initial concentration of 10^6^ colony-forming units (CFUs) per mL, and mixed in a 4:1 ratio (attacker: prey). Next, the mixture was divided into two parts. One hundred microliters of this mixture was inoculated in triplicate onto 0.22 µm nitrocellulose membranes, which were placed on LB plates. After 4 h of incubation at 37°C, bacterial cells were collected from the membranes, and 10-fold serial dilutions were plated on LB plates containing gentamicin (30 µg/mL) to evaluate the survival of *E. coli* and quantify their CFU. The remaining portion of the mixture was subjected to a 10-fold serial dilution and incubated on selective medium overnight to determine the initial concentration of *E. coli*. Following the same procedures, two sets of bacterial fluids were plated on LB plates containing ampicillin (75 µg/mL, to which *V. parahaemolyticus* is resistant) and were used to determine the initial and post-competitive viability counts of the attackers. This experiment was repeated at least three times.

### Biofilm formation assay

The crystal violet staining assay was adapted from a previously described protocol (54). Briefly, exponential-phase bacterial cultures were diluted 1:100 in fresh 3% NaCl-LB medium, and 200 µL aliquots were transferred to sterile 96-well flat-bottomed plates. Plates were incubated at 37°C or 30°C for 48 h, after which wells were washed with PBS and stained with 200 µL of 1% crystal violet for 15 min. Unbound dye was removed by PBS washing, and bound crystal violet was solubilized in 200 µL of 95% (vol/vol) ethanol. Absorbance was measured at 595 nm using a spectrophotometer (Thermo Fisher, Finland). All assays were performed in triplicate, and the experiment was repeated three times with new cultures.

### Motility assay

Motility assays were performed as previously described (55). For the swimming motility assay, 2 µL of bacterial culture in logarithmic growth phase was inoculated onto the surface of LB plates containing 1% NaCl and 0.3% agar. Each plate was then incubated at 37°C for 5 h, after which the colony diameter was measured and recorded. For the swarming motility assay, the strains were inoculated onto BHI plates containing 2% NaCl and 1.5% agar and incubated at 30°C for 15–20 h, after which swarm diameters were documented.

### Animal infection experiments

Three-week-old ICR mice were randomly divided into seven groups: WT, Δ*vipA1*, Δ*hcp1*, Δ*vipA1-hcp1*, CΔ*vipA1*, CΔ*hcp1*, and a blank control group, with eight mice per group. Following the method described by Hiyoshi et al. (56), each bacterial strain was grown to the logarithmic stage, washed three times with sterile saline, and adjusted to a concentration of 1 × 10^5^ CFU/mL. Subsequently, 100 µL of the bacterial solution was injected intraperitoneally in the respective test group, while the blank group received an equivalent dose of sterile saline. The survival rate of the ICR mice in each group was monitored and recorded hourly for 7 consecutive days. A survival curve was plotted for each strain, with time serving as the horizontal axis and the survival rate of mice in each group as the vertical axis.

The ability of bacteria to colonize and proliferate was assessed in a systemic infection model. Ten mice were intravenously inoculated with a bacterial suspension containing 1 × 10⁷ CFU. At 20 h postinfection, blood, spleens, and livers were harvested for analysis. Tissue homogenates were serially diluted and plated onto LB agar plates supplemented with 3% NaCl to quantify bacterial load and evaluate colonization efficiency.

Furthermore, this study examined and compared the histopathological changes in the livers and spleens of 3-week-old mice intravenously administered 1 × 10⁷ CFU under sedation, with those observed in the control group. At 20 h postinfection, organs from each group were harvested, fixed in 4% neutral-buffered formalin, and then sent to the Shanghai Experimental Animal Center for histopathological evaluation. Tissues from uninfected mice were used as negative controls for this comparative analysis.

### Cytotoxicity assays

To assess bacteria-mediated cytotoxicity, we measured the release of lactate dehydrogenase (LDH) from the supernatants of HeLa cell cultures. All bacterial strains were prepared as previously described (57). Prior to infection, the cells were washed with PBS at pH 7.2 and then incubated with DMEM without phenol red. Bacterial suspensions were added to 96-well plates at an infection ratio of 10:1, and the plates were centrifuged at 600 × *g* for 10 min to synchronize the infection. Following a 2 h incubation, the cell culture supernatants were collected and analyzed for LDH release using the CytoTox 96 Assay Kit (Promega, Madison, WI) according to the manufacturer’s instructions. The experiment was performed in triplicate.

### Cytokine assays

HeLa cells were cultivated in 24-well cell culture plates until reaching 80%–90% confluence. Each bacterial strain was inoculated at an infection ratio of 10:1. After 1 h of infection, the cells were treated with DMEM containing 100 µg/mL gentamicin. RNA and protein samples were collected at 1 h, 2 h, 4 h, and 8 h post-infection. The cells were incubated for the indicated durations to allow for the collection of both cellular and supernatant protein samples. Cellular mRNA was extracted using the TRIzol method and subsequently transcribed into cDNA using the HiScript II 1st Strand cDNA Synthesis Kit (+gDNA wiper).

### Detection of key proteins in the NF-κB pathway

HeLa cells were infected with each strain for 1 h, after which the infection was terminated by adding DMEM containing 100 µg/mL gentamicin and 10 ng/mL IL-1β. This point was recorded as 0 min, and protein samples were collected at 60 and 90 min post-termination. Following cell lysis, 5× protein sample buffer was added. They were subsequently separated on SDS-PAGE, transferred to a polyvinylidene difluoride membrane, and immunoblotted using specific antibodies against IκBα, Phospho-IκBα, NF-κB p65, Phospho-NF-κB p65, or β-actin (from CST, Beverly, MA, USA) at a 1:1,000 dilution. Protein signals were visualized using enhanced chemiluminescence.

### p65 translocation assays

The p65 translocation assays were conducted according to the methodology outlined in reference (51). HeLa cells were inoculated into 24-well cell culture plates with preformed cell monolayers. Subsequently, the cells were infected with different strains at an MOI ratio of 10:1 for 1 h. After the infection period, the infection was terminated by adding blank DMEM containing 50 µg/mL gentamicin and 10 ng/mL IL-1β. The cells were stimulated with IL-1β for 1 h, washed three times with PBS. They were then fixed with 4% paraformaldehyde for 10 min at room temperature and subsequently washed three times with PBS. The cells were permeabilized with 0.05% Triton X-100 for 10 min at room temperature and washed twice with PBS. Blocking was carried out using 3% bovine serum albumin, after which the cells were incubated with the p65 primary antibody and subsequently labeled with Alexa Fluor 488 antibody (Abcam). The nuclei were stained with DAPI to visualize DNA, and images were captured using a Zeiss confocal microscope.

### Statistical analyses

All experiments were repeated at least three times, and one-way analysis of variance (ANOVA), Student’s *t*-test, and two-way ANOVA were performed using GraphPad Prism 9.5 (GraphPad Software Inc.). Statistical analysis indicated a significant difference (ns: not significant; *, *P* < 0.05; **, *P* < 0.01; ***, *P* < 0.001). Protein blot bands were quantified and analyzed using ImageJ statistical software.

## SUPPLEMENTAL MATERIAL

Fig. S1 iai.00587-25-s0001.docx The growth curve of the wild-type (WT), mutant strain (Δ*vipA1*, Δ*hcp1*, and Δ*vipA1*-*hcp1*), and complement strain (CΔ*vipA1* and CΔ*hcp1*).

Fig. S2 iai.00587-25-s0002.docx (A) GO terms for the WT and Δ*vipA1*-*hcp1* groups and (B) GO terms for the WT and Δ*hcp1* groups. (C) KEGG pathway analysis of DEPs in WT and Δ*vipA1*-*hcp1* (D) KEGG pathway analysis of DEPs in WT and Δ*hcp1*.

Table S1 iai.00587-25-s0003.docx Primers used in this study.

Table S2 iai.00587-25-s0004.docx qRT-PCR primers.

ASM does not own the copyrights to Supplemental Material that may be linked to, or accessed through, an article. The authors have granted ASM a non-exclusive, world-wide license to publish the Supplemental Material files. Please contact the corresponding author directly for reuse.

## References

[1] Ashrafudoulla M, Na KW, Hossain MI, Mizan MFR, Nahar S, Toushik SH, Roy PK, Park SH, Ha S-D. 2021. Molecular and pathogenic characterization of Vibrio parahaemolyticus isolated from seafood. Mar Pollut Bull 172:112927. doi:10.1016/j.marpolbul.2021.112927 34526263

[2] Shimohata T, Takahashi A. 2010. Diarrhea induced by infection of Vibrio parahaemolyticus. J Med Invest 57:179–182. doi:10.2152/jmi.57.179 20847516

[3] Ahmad JN, Sebo P. 2021. Bacterial RTX toxins and host immunity. Curr Opin Infect Dis 34:187–196. doi:10.1097/QCO.0000000000000726 33899753

[4] Basler M, Ho BT, Mekalanos JJ. 2013. Tit-for-tat: type VI secretion system counterattack during bacterial cell-cell interactions. Cell 152:884–894. doi:10.1016/j.cell.2013.01.042 23415234 PMC3616380

[5] Basler M, Pilhofer M, Henderson GP, Jensen GJ, Mekalanos JJ. 2012. Type VI secretion requires a dynamic contractile phage tail-like structure. Nature 483:182–186. doi:10.1038/nature10846 22367545 PMC3527127

[6] Clemens DL, Ge P, Lee B-Y, Horwitz MA, Zhou ZH. 2015. Atomic structure of T6SS reveals interlaced array essential to function. Cell 160:940–951. doi:10.1016/j.cell.2015.02.005 25723168 PMC4351867

[7] Kube S, Kapitein N, Zimniak T, Herzog F, Mogk A, Wendler P. 2014. Structure of the VipA/B type VI secretion complex suggests a contraction-state-specific recycling mechanism. Cell Rep 8:20–30. doi:10.1016/j.celrep.2014.05.034 24953649

[8] Silverman JM, Agnello DM, Zheng H, Andrews BT, Li M, Catalano CE, Gonen T, Mougous JD. 2013. Haemolysin coregulated protein is an exported receptor and chaperone of type VI secretion substrates. Mol Cell 51:584–593. doi:10.1016/j.molcel.2013.07.025 23954347 PMC3844553

[9] Ahmad S, Wang B, Walker MD, Tran H-K, Stogios PJ, Savchenko A, Grant RA, McArthur AG, Laub MT, Whitney JC. 2019. An interbacterial toxin inhibits target cell growth by synthesizing (p)ppApp. Nature 575:674–678. doi:10.1038/s41586-019-1735-9 31695193 PMC6883173

[10] Russell AB, Wexler AG, Harding BN, Whitney JC, Bohn AJ, Goo YA, Tran BQ, Barry NA, Zheng H, Peterson SB, Chou S, Gonen T, Goodlett DR, Goodman AL, Mougous JD. 2014. A type VI secretion-related pathway in Bacteroidetes mediates interbacterial antagonism. Cell Host Microbe 16:227–236. doi:10.1016/j.chom.2014.07.007 25070807 PMC4136423

[11] Kapitein N, Bönemann G, Pietrosiuk A, Seyffer F, Hausser I, Locker JK, Mogk A. 2013. ClpV recycles VipA/VipB tubules and prevents non-productive tubule formation to ensure efficient type VI protein secretion. Mol Microbiol 87:1013–1028. doi:10.1111/mmi.12147 23289512

[12] Pazhani GP, Chowdhury G, Ramamurthy T. 2021. Adaptations of Vibrio parahaemolyticus to stress during environmental survival, host colonization, and infection. Front Microbiol 12:737299. doi:10.3389/fmicb.2021.737299 34690978 PMC8530187

[13] Yu Y, Yang H, Li J, Zhang P, Wu B, Zhu B, Zhang Y, Fang W. 2012. Putative type VI secretion systems of Vibrio parahaemolyticus contribute to adhesion to cultured cell monolayers. Arch Microbiol 194:827–835. doi:10.1007/s00203-012-0816-z 22535222

[14] Cohen H, Baram N, Fridman CM, Edry-Botzer L, Salomon D, Gerlic M. 2022. Post-phagocytosis activation of NLRP3 inflammasome by two novel T6SS effectors. eLife 11:e82766. doi:10.7554/eLife.82766 36155655 PMC9545535

[15] Monjarás Feria J, Valvano MA. 2020. An overview of anti-eukaryotic T6SS effectors. Front Cell Infect Microbiol 10:584751. doi:10.3389/fcimb.2020.584751 33194822 PMC7641602

[16] Krakauer T. 2019. Inflammasomes, autophagy, and cell death: the trinity of innate host defense against intracellular bacteria. Mediators Inflamm 2019:2471215. doi:10.1155/2019/2471215 30728749 PMC6341260

[17] Sá-Pessoa J, López-Montesino S, Przybyszewska K, Rodríguez-Escudero I, Marshall H, Ova A, Schroeder GN, Barabas P, Molina M, Curtis T, Cid VJ, Bengoechea JA. 2023. A trans-kingdom T6SS effector induces the fragmentation of the mitochondrial network and activates innate immune receptor NLRX1 to promote infection. Nat Commun 14:871. doi:10.1038/s41467-023-36629-3 36797302 PMC9935632

[18] Salomon D, Gonzalez H, Updegraff BL, Orth K. 2013. Vibrio parahaemolyticus type VI secretion system 1 is activated in marine conditions to target bacteria, and is differentially regulated from system 2. PLoS One 8:e61086. doi:10.1371/journal.pone.0061086 23613791 PMC3628861

[19] Bönemann G, Pietrosiuk A, Diemand A, Zentgraf H, Mogk A. 2009. Remodelling of VipA/VipB tubules by ClpV-mediated threading is crucial for type VI protein secretion. EMBO J 28:315–325. doi:10.1038/emboj.2008.269 19131969 PMC2646146

[20] Zhang N, Ye F, Wang Y, Liu R, Huang Z, Chen C, Liu L, Kang X, Dong S, Rajaofera MJN, Zhu C, Zhang L, Zhou Y, Xiong Y, Xia Q. 2023. Role of type VI secretion system protein TssJ-3 in virulence and intracellular survival of Burkholderia pseudomallei. Biochem Biophys Res Commun 682:397–406. doi:10.1016/j.bbrc.2023.09.091 37852065

[21] Lucchini V, Sivignon A, Pieren M, Gitzinger M, Lociuro S, Barnich N, Kemmer C, Trebosc V. 2021. The role of OmpR in bile tolerance and pathogenesis of adherent-invasive Escherichia coli. Front Microbiol 12:684473. doi:10.3389/fmicb.2021.684473 34262546 PMC8273539

[22] Yu F, Dong C, Zhang Y, Che R, Xie C, Liu Y, Zhang Z, Li L, Chen X, Cai X, Wang G, Li Y. 2023. GrpE and ComD contribute to the adherence, biofilm formation, and pathogenicity of Streptococcus suis. Arch Microbiol 205:159. doi:10.1007/s00203-023-03503-1 37005968

[23] Lu G-T, Tang Y-Q, Li C-Y, Li R-F, An S-Q, Feng J-X, He Y-Q, Jiang B-L, Tang D-J, Tang J-L. 2009. An adenosine kinase exists in Xanthomonas campestris pathovar campestris and is involved in extracellular polysaccharide production, cell motility, and virulence. J Bacteriol 191:3639–3648. doi:10.1128/JB.00009-09 19329636 PMC2681908

[24] Fridman CM, Jana B, Ben-Yaakov R, Bosis E, Salomon D. 2022. A DNase type VI Secretion system effector requires its MIX domain for secretion. Microbiol Spectr 10:e0246522. doi:10.1128/spectrum.02465-22 36098406 PMC9602870

[25] Wang T, Du X, Ji L, Han Y, Dang J, Wen J, Wang Y, Pu Q, Wu M, Liang H. 2021. Pseudomonas aeruginosa T6SS-mediated molybdate transport contributes to bacterial competition during anaerobiosis. Cell Rep 35:108957. doi:10.1016/j.celrep.2021.108957 33852869

[26] Soria-Bustos J, Ares MA, Gómez-Aldapa CA, González-Y-Merchand JA, Girón JA, De la Cruz MA. 2020. Two type VI secretion systems of Enterobacter cloacae are required for bacterial competition, cell adherence, and intestinal colonization. Front Microbiol 11:560488. doi:10.3389/fmicb.2020.560488 33072020 PMC7541819

[27] Silva AJ, Benitez JA. 2016. Vibrio cholerae biofilms and cholera pathogenesis. PLoS Negl Trop Dis 10:e0004330. doi:10.1371/journal.pntd.0004330 26845681 PMC4741415

[28] Shiba T, Tsutsumi K, Yano H, Ihara Y, Kameda A, Tanaka K, Takahashi H, Munekata M, Rao NN, Kornberg A. 1997. Inorganic polyphosphate and the induction of rpoS expression. Proc Natl Acad Sci USA 94:11210–11215. doi:10.1073/pnas.94.21.11210 9326588 PMC23418

[29] Ringgaard S, Hubbard T, Mandlik A, Davis BM, Waldor MK. 2015. RpoS and quorum sensing control expression and polar localization of Vibrio cholerae chemotaxis cluster III proteins in vitro and in vivo. Mol Microbiol 97:660–675. doi:10.1111/mmi.13053 25989366 PMC4646612

[30] Chen W, Palmer RJ, Kuramitsu HK. 2002. Role of polyphosphate kinase in biofilm formation by Porphyromonas gingivalis. Infect Immun 70:4708–4715. doi:10.1128/IAI.70.8.4708-4715.2002 12117989 PMC128176

[31] Yepes A, Schneider J, Mielich B, Koch G, García-Betancur J-C, Ramamurthi KS, Vlamakis H, López D. 2012. The biofilm formation defect of a Bacillus subtilis flotillin-defective mutant involves the protease FtsH. Mol Microbiol 86:457–471. doi:10.1111/j.1365-2958.2012.08205.x 22882210 PMC3988463

[32] Bouillet S, Bauer TS, Gottesman S. 2024. RpoS and the bacterial general stress response. Microbiol Mol Biol Rev 88:e0015122. doi:10.1128/mmbr.00151-22 38411096 PMC10966952

[33] Fu D, Wu J, Gu Y, Li Q, Shao Y, Feng H, Song X, Tu J, Qi K. 2022. The response regulator OmpR contributes to the pathogenicity of avian pathogenic Escherichia coli. Poult Sci 101:101757. doi:10.1016/j.psj.2022.101757 35240350 PMC8892008

[34] de Pace F, Boldrin de Paiva J, Nakazato G, Lancellotti M, Sircili MP, Guedes Stehling E, Dias da Silveira W, Sperandio V. 2011. Characterization of IcmF of the type VI secretion system in an avian pathogenic Escherichia coli (APEC) strain. Microbiology (Reading) 157:2954–2962. doi:10.1099/mic.0.050005-0 21778203 PMC3353391

[35] Das S, Chakrabortty A, Banerjee R, Chaudhuri K. 2002. Involvement of in vivo induced icmF gene of Vibrio cholerae in motility, adherence to epithelial cells, and conjugation frequency. Biochem Biophys Res Commun 295:922–928. doi:10.1016/s0006-291x(02)00782-9 12127983

[36] Liu L, Hao S, Lan R, Wang G, Xiao D, Sun H, Xu J. 2015. The type VI secretion system modulates flagellar gene expression and secretion in Citrobacter freundii and contributes to adhesion and cytotoxicity to host cells. Infect Immun 83:2596–2604. doi:10.1128/IAI.03071-14 25870231 PMC4468558

[37] Huang L, Guo L, Xu X, Qin Y, Zhao L, Su Y, Yan Q. 2019. The role of rpoS in the regulation of Vibrio alginolyticus virulence and the response to diverse stresses. J Fish Dis 42:703–712. doi:10.1111/jfd.12972 30811044

[38] Kamal SM, Rybtke ML, Nimtz M, Sperlein S, Giske C, Trček J, Deschamps J, Briandet R, Dini L, Jänsch L, Tolker-Nielsen T, Lee C, Römling U. 2019. Two FtsH proteases contribute to fitness and adaptation of Pseudomonas aeruginosa clone C strains. Front Microbiol 10:1372. doi:10.3389/fmicb.2019.01372 31338071 PMC6629908

[39] Wang W, Jiang J, Chen H, Zhang Y, Liu Q. 2021. FtsH is required for protein secretion homeostasis and full bacterial virulence in Edwardsiella piscicida. Microb Pathog 161:105194. doi:10.1016/j.micpath.2021.105194 34534640

[40] Ishikawa T, Sabharwal D, Bröms J, Milton DL, Sjöstedt A, Uhlin BE, Wai SN. 2012. Pathoadaptive conditional regulation of the type VI secretion system in Vibrio cholerae O1 strains. Infect Immun 80:575–584. doi:10.1128/IAI.05510-11 22083711 PMC3264300

[41] Bröms JE, Ishikawa T, Wai SN, Sjöstedt A. 2013. A functional VipA-VipB interaction is required for the type VI secretion system activity of Vibrio cholerae O1 strain A1552. BMC Microbiol 13:96. doi:10.1186/1471-2180-13-96 23642157 PMC3656785

[42] Wang P, Dong J-F, Li R-Q, Li L, Zou Q-H. 2020. Roles of the Hcp family proteins in the pathogenicity of Salmonella typhimurium 14028s. Virulence 11:1716–1726. doi:10.1080/21505594.2020.1854538 33300449 PMC7733977

[43] Tu J, Shen X, Chen Z, Hou M, Song Z, Jiang H, Shao Y, Qi K, Song X. 2022. Hcp2b of T6SS affects colonization of avian pathogenic Escherichia coli and host keratin filament expression. Avian Pathol 51:154–163. doi:10.1080/03079457.2022.2031881 35076320

[44] Osorio CR, Juiz-Río S, Lemos ML. 2010. The ABC-transporter hutCD genes of Photobacterium damselae subsp. piscicida are essential for haem utilization as iron source and are expressed during infection in fish. J Fish Dis 33:649–655. doi:10.1111/j.1365-2761.2010.01169.x 20561140

[45] Cai Y, Dong J, Huang J, He J, Hu Y, Sui Z, Tang P. 2024. The cyclic AMP (cAMP) phosphodiesterase CpdA required for growth, biofilm formation, motility and pathogenicity of Edwardsiella piscicida. Microb Pathog 188:106545. doi:10.1016/j.micpath.2024.106545 38244636

[46] da Silva AA, Galego L, Arraiano CM. 2023. New perspectives on BolA: a still mysterious protein connecting morphogenesis, biofilm production, virulence, iron metabolism, and stress survival. Microorganisms 11:632. doi:10.3390/microorganisms11030632 36985206 PMC10051749

[47] Mil-Homens D, Barahona S, Moreira RN, Silva IJ, Pinto SN, Fialho AM, Arraiano CM. 2018. Stress response protein BolA influences fitness and promotes Salmonella enterica serovar Typhimurium virulence. Appl Environ Microbiol 84:e02850-17. doi:10.1128/AEM.02850-17 29439986 PMC5881071

[48] Cai H, Yu J, Qiao Y, Ma Y, Zheng J, Lin M, Yan Q, Huang L. 2022. Effect of the type VI secretion system secreted protein Hcp on the virulence of Aeromonas salmonicida. Microorganisms 10:2307. doi:10.3390/microorganisms10122307 36557560 PMC9784854

[49] Coulthurst SJ. 2013. The type VI secretion system - a widespread and versatile cell targeting system. Res Microbiol 164:640–654. doi:10.1016/j.resmic.2013.03.017 23542428

[50] Bönemann G, Pietrosiuk A, Mogk A. 2010. Tubules and donuts: a type VI secretion story. Mol Microbiol 76:815–821. doi:10.1111/j.1365-2958.2010.07171.x 20444095

[51] Zhou X, Gewurz BE, Ritchie JM, Takasaki K, Greenfeld H, Kieff E, Davis BM, Waldor MK. 2013. A Vibrio parahaemolyticus T3SS effector mediates pathogenesis by independently enabling intestinal colonization and inhibiting TAK1 activation. Cell Rep 3:1690–1702. doi:10.1016/j.celrep.2013.03.039 23623501 PMC3711673

[52] Park K-S, Ono T, Rokuda M, Jang M-H, Okada K, Iida T, Honda T. 2004. Functional characterization of two type III secretion systems of Vibrio parahaemolyticus. Infect Immun 72:6659–6665. doi:10.1128/IAI.72.11.6659-6665.2004 15501799 PMC523034

[53] Lian L, Xue J, Li W, Ren J, Tang F, Liu Y, Xue F, Dai J. 2021. VscF in T3SS1 helps to translocate VPA0226 in Vibrio parahaemolyticus. Front Cell Infect Microbiol 11:652432. doi:10.3389/fcimb.2021.652432 33869083 PMC8047418

[54] Zhang Y, Qiu Y, Xue X, Zhang M, Sun J, Li X, Hu L, Yin Z, Yang W, Lu R, Zhou D. 2021. Transcriptional regulation of the virulence genes and the biofilm formation associated operons in Vibrio parahaemolyticus. Gut Pathog 13:15. doi:10.1186/s13099-021-00410-y 33653369 PMC7923509

[55] Whitaker WB, Richards GP, Boyd EF. 2014. Loss of sigma factor RpoN increases intestinal colonization of Vibrio parahaemolyticus in an adult mouse model. Infect Immun 82:544–556. doi:10.1128/IAI.01210-13 24478070 PMC3911383

[56] Hiyoshi H, Kodama T, Iida T, Honda T. 2010. Contribution of Vibrio parahaemolyticus virulence factors to cytotoxicity, enterotoxicity, and lethality in mice. Infect Immun 78:1772–1780. doi:10.1128/IAI.01051-09 20086084 PMC2849405

[57] Li Y, Sun W, Wang Q, Yu Y, Wan Y, Zhou K, Guo R, Han X, Chen Z, Fang W, Jiang W. 2022. The GntR-like transcriptional regulator HutC involved in motility, biofilm-forming ability, and virulence in Vibrio parahaemolyticus. Microb Pathog 167:105546. doi:10.1016/j.micpath.2022.105546 35512440

