# Supplementary material for: T6SS1 suppresses pro-inflammatory cytokine transcription to drive immune evasion and systemic infection in Vibrio parahaemolyticus
Source: Infect Immun. 2025 Dec 5;94(1):e00587-25. doi: 10.1128/iai.00587-25 (PMC12797937; doi:10.1128/iai.00587-25)
Supplement: Fig. S1 — The growth curve of the wild-type (WT), mutant strain (ΔvipA1, Δhcp1, and ΔvipA1-hcp1), and complement strain (CΔvipA1 and CΔhcp1). [file iai.00587-25-s0001.docx]

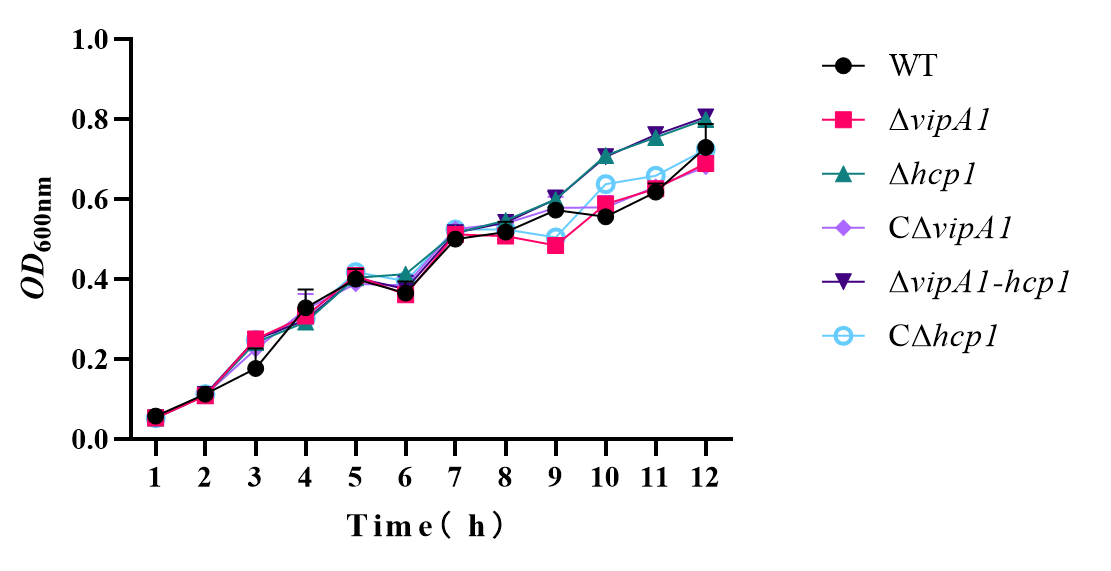


**Supplementary material Fig S1** The growth curve of the wild-type (WT)，mutant strain (Δ*vipA1,* Δ*hcp1,* Δ*vipA1-hcp1*) and complement strain(CΔ*vipA1,* CΔ*hcp1*).
