# Supplementary material for: T6SS1 suppresses pro-inflammatory cytokine transcription to drive immune evasion and systemic infection in Vibrio parahaemolyticus
Source: Infect Immun. 2025 Dec 5;94(1):e00587-25. doi: 10.1128/iai.00587-25 (PMC12797937; doi:10.1128/iai.00587-25)
Supplement: Fig. S2 — (A) GO terms for the WT and ΔvipA1-hcp1 groups and (B) GO terms for the WT and Δhcp1 groups. (C) KEGG pathway analysis of DEPs in WT and ΔvipA1-hcp1 (D) KEGG pathway analysis of DEPs in WT and Δhcp1. [file iai.00587-25-s0002.docx]

A


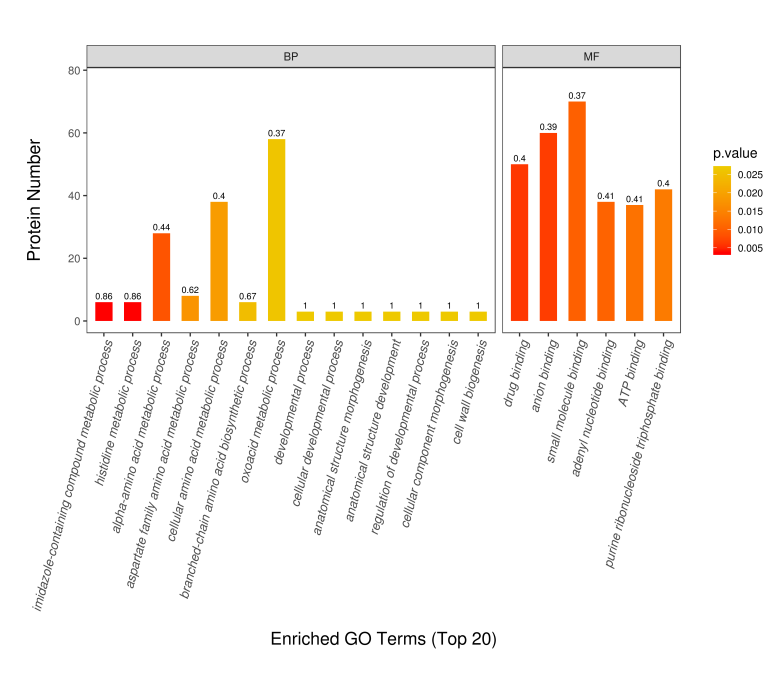

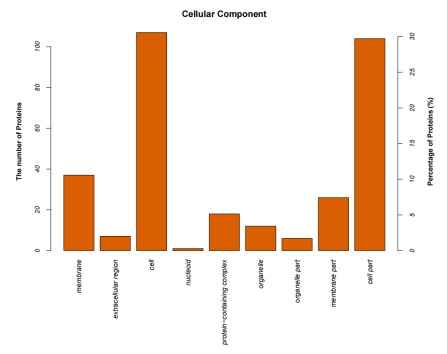

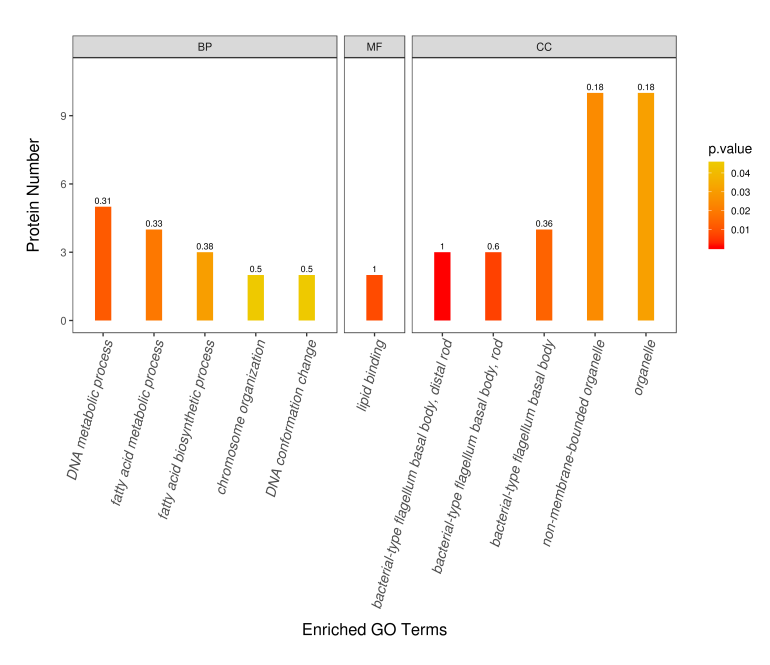


B

C


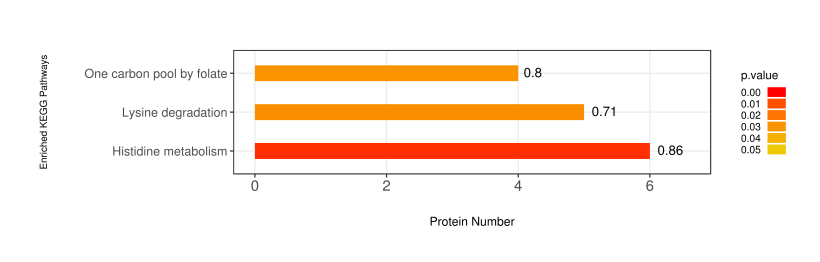

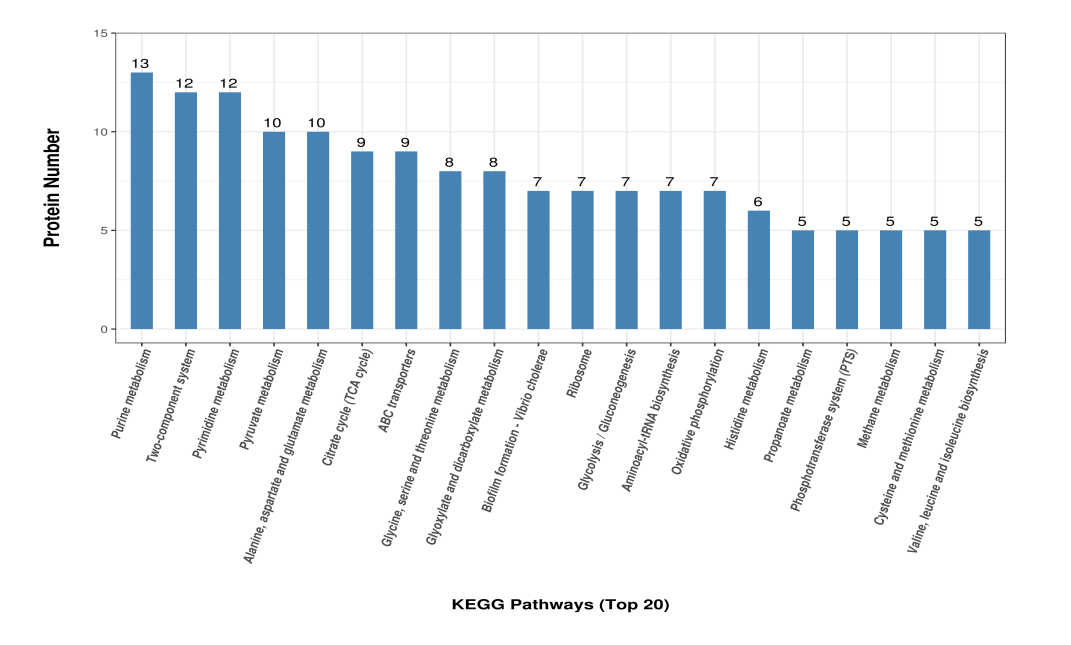

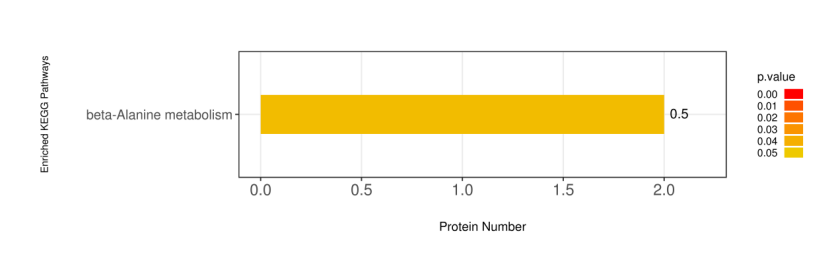

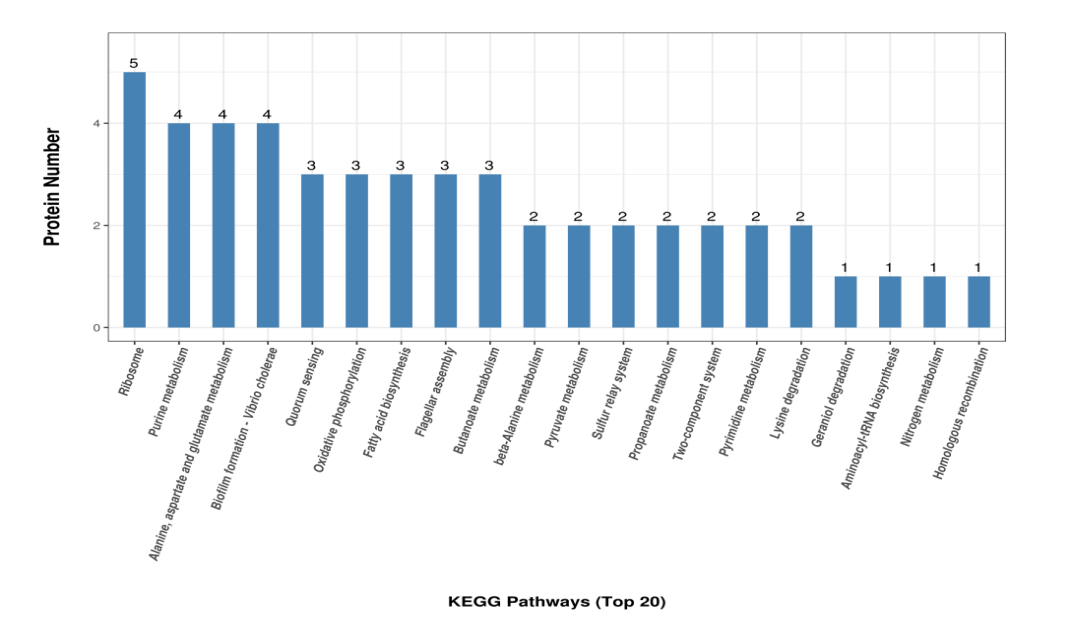


D

**Supplementary material Fig. S2** (A) GO terms for the WT and Δ*vipA1-hcp1* groups and (B) GO terms for the WT and Δ*hcp1* groups; (C) KEGG pathway analysis of DEPs in WT and Δ*vipA1-hcp1* (D) KEGG pathway analysis of DEPs in WT and Δ*hcp1*. Bar colors represent a logarithmic scale from -2 to 2.
