# Supplementary material for: T6SS1 suppresses pro-inflammatory cytokine transcription to drive immune evasion and systemic infection in Vibrio parahaemolyticus
Source: Infect Immun. 2025 Dec 5;94(1):e00587-25. doi: 10.1128/iai.00587-25 (PMC12797937; doi:10.1128/iai.00587-25)
Supplement: Table S1 — Primers used in this study. [file iai.00587-25-s0003.docx]

Supplementary material Table S1 Primers used in this study

| Primer | Sequence (5’to 3’)^a^ | Restriction site | Target gene |
| --- | --- | --- | --- |
| *vipA1*-A | AAAGGATCCGCTTCAAGTGCTCAAT | *Bam* HI | Updream region of *vipA1* ORF (416 bp) |
| *vipA1*-B | AATTAGTTCCTTATCTAACTCTTTAATTAGTCAT |  |  |
| *vipA1*-C | GATAAGGAACTAATTGGGATATCATGTCTGCA |  | Down dream region of *vipA1* ORF（373 bp） |
| *vipA1*-D | AATGCATGCTTTGAATGTTCTCACGGA | *Sph* I |  |
| *vipA1*-E | GTCACCTAACTTGGGATG |  | This primer was used to confirm the deletion of *vipA1*.  Wide type: 1770bp;  Mutant: 1263bp |
| *vipA1*-F | ACAGATTACCGCACCTAC |  |  |
| *vipA1*-pMMB-F | ACAGAATTCATGTCACGTGACGGCTCGGT | *Eco* RI | A fragment for complementation of *vipA1* (525bp) |
| *vipA1*-pMMB-R | TCTGGATCCTTACTCTTCTTTCGCGTCTTGGT | *Bam* HI |  |
| *vipA1*-pET28-F | TTTGGATCCATGGGCAGTTCGTCTAT | *Bam* HI | A fragment for expression of *vipA1 (*525bp*)* |
| *vipA1*-pET28-R | CCTGAGCTCTTATCGATACACTTCA | *Sac* I |  |
| *hcp1*-A | AAAGGATCCACGACTAAAATGCCAGTGTTA | *Bam* HI | Updream region of *hcp1* ORF (287 bp) |
| *hcp1*-B | CGCTATTTCCTTTTCTAAAATCTGTTTTGTT |  |  |
| *hcp1*-C | GAAAAGGAAATAGCGTTGCTTTTTGCGTAAAGATTCA |  | Down dream region of *hcp1* ORF（431 bp） |
| *hcp1*-D | AATGCATGCTGTTGGAAAATACGACAGTCT | *Sph* I |  |
| *hcp1*-E | TGGATCGAACCGATTGTCTC |  | This primer was used to confirm the deletion of *hcp1.*  Wide type: 1992bp  Mutant: 1473bp |
| *hcp1*-F | TGGCTGCTCGTAGTTGTAGT |  |  |
| *hcp1*-pMMB-F | CCTGAATTCATGCCAACTCCAGCATATATGTCA | *Eco* RI | A fragment for complementation of *hcp1* |
| *hcp1*-pMMB-R | ATTGGATCCTTATGCTTCGCGTGGAGCA | *Bam* HI |  |
| *hcp1*-pET28-F | CCAGGATCCatgccaactccagc | *Bam* HI | A fragment for expression of *hcp1* |
| *hcp1*-pET28-R | GCAGAGCTCttaagcttcgcgtgga | *Sac* I |  |

^a^ Restriction sites are underlined.
