# Supplementary material for: T6SS1 suppresses pro-inflammatory cytokine transcription to drive immune evasion and systemic infection in Vibrio parahaemolyticus
Source: Infect Immun. 2025 Dec 5;94(1):e00587-25. doi: 10.1128/iai.00587-25 (PMC12797937; doi:10.1128/iai.00587-25)
Supplement: Table S2 — qRT-PCR primers. [file iai.00587-25-s0004.docx]

| Primer name | | Sequence (5′→3′) | Source or reference |
| --- | --- | --- | --- |
| RT-IL-6-F | GGCACTGGCAGAAAACAAC | | This study |
| RT-IL-6-R | GCAAGTCTCCTCATTGAATC | | This study |
| RT-IL-1β-F | CGAATCTCCGACCACCACTA | | This study |
| RT-IL-1β-R | GCACATAAGCCTCGTTATCCC | | This study |
| RT-IL-8-F | TTGGCAGCCTTCCTGATTT' | | This study |
| RT-IL-8-R | GGGGTGGAAAGGTTTGGAG | | This study |
| RT-β-actin-F | CACTCTTCCAGCCTTCCTTCC | | This study |
| RT-β-actin-R | CAGGTCTTTGCGGATGTCCA | | This study |

Supplementary material Table S2 qRT-PCR primers
